# The Effect of S616L, a CPVT1-Associated Mutation, on the Stability and Structure of Human Cardiac RyR2

**DOI:** 10.3390/ijms272110052

**Published:** 2026-11-09

**Authors:** Jacob A. Bauer, Mário Benko, Ľubica Šutová, Konrad Beck, Jakub Nowak, Jana Bellová, Peter Baráth, Vladena Bauerová-Hlinková

**Affiliations:** 1 Institute of Molecular Biology, Slovak Academy of Sciences, Dúbravská Cesta 21, 845 51 Bratislava, Slovakia; mario.benko@savba.sk (M.B.); lubica.macakova94@gmail.com (Ľ.Š.); vladena.bauerova@savba.sk (V.B.-H.); 2 School of Dentistry, Cardiff University, Heath Park, Cardiff CF14 4XY, UK; 3 Nanotemper Technologies, 30-348 Krakow, Poland; kuba.nowak@uj.edu.pl; 4 Institute of Chemistry, Slovak Academy of Sciences, Dúbravská Cesta 9, 845 38 Bratislava, Slovakia; jana.bellova@savba.sk (J.B.); peter.barath@savba.sk (P.B.)

**Keywords:** human ryanodine receptor 2 (*h*RyR2), catecholaminergic polymorphic ventricular tachycardia type 1 (CPVT1), CD spectroscopy, nanoDSF, microscale thermophoresis, mutation

## Abstract

Catecholaminergic polymorphic ventricular tachycardia type 1 (CPVT1) is a heritable genetic disease caused by mutations to *h*RyR2, the cardiac isoform of the human ryanodine receptor. One such mutation, S616L, is found in the α-helical part (NSol) of the *h*RyR2 N-terminal domain (NTD) close to a three-way junction between the NTD, junctional solenoid (JSol), and bridging solenoid (BSol) domains. In this study, we use molecular dynamics simulation to study the effect of S616L on the residues of this junction. We find that the mutation slightly weakens the interface between the N-terminal domain and the bridging solenoid by changing the preferred conformation of the residues at the interface, thereby promoting a slight opening between these domains. In addition, we determined the impact of this mutation on the biophysical and biochemical properties of a 655-residue fragment containing the entire *h*RyR2 N-terminal domain, finding that it slightly increases the thermal stability of the fragment.

## 1. Introduction

The ryanodine receptor (RyR) is the principal Ca^2+^ release channel in muscle cells [1,2]. In humans, three different isoforms of RyR are found: *h*RyR1 occurs most often in skeletal muscle cells [3], *h*RyR2 appears predominantly in cardiac tissue [4,5], and *h*RyR3 appears in a variety of tissues including, the brain and smooth muscle tissue [6,7]. In all these tissues, RyR is found embedded in the membrane of the sarcoplasmic or endoplasmic reticulum where it opens in response to a particular signal in order to release the stored Ca^2+^ into the cytosol. Normally, opening is triggered by a local increase in Ca^2+^ concentration; in muscle cells, this is provided by voltage-gated calcium channels, which are located opposite RyR in the cytoplasmic membrane. Closing is triggered by the binding of Ca^2+^ to low-affinity Ca^2+^-binding sites once the local concentration reaches a suitable threshold [8].

Many cryo-EM structures of the complete RyR1 and RyR2 from a number of organisms have been determined, allowing this channel to be described structurally [9,10,11,12,13,14,15]. The *h*RyR2 channel can be described as a homotetramer with a rough square pyramid shape. Each monomer has approximately 5000 residues divided into ten distinct domains: an N-terminal domain (NTD; residues 1–640, *h*RyR2 numbering), three SPRY domains (SPRY1: 641–837, 856–861, 1463–1484, 1601–1641; SPRY2: 838–856, 1073–1254, 1595–1600; SPRY3: 1255–1462, 1485–1594), interrupted by the RY12 repeat domain (residues 862–1072), a JSol (junctional solenoid) domain (residues 1642–2110), a single long bridging α-helical solenoid (BSol) interrupted in sequence but not in structure by RY34 (BSol1: 2111–2683; RY34: 2684–2911; BSol2: 2912–3564), a core solenoid (CSol) domain (3635–4209), and the transmembrane Ion Channel domain (4237–4968).

The RyR is regulated by a wide variety of factors [16,17], and its dysfunction can lead to a number of heritable genetic diseases depending on which isoform is mutated [2]. For example, *h*RyR1 dysfunction most frequently causes malignant hyperthermia [18,19], central core disease [20], and multi-minicore disease [21]. *h*RyR2 dysfunction is associated with catecholaminergic polymorphic ventricular tachycardia, type 1 (CPVT1) [22,23], and arrhythmogenic right ventricular cardiomyophathy/dysplasia, type 2 (ARVC/D2) [24]. There is evidence suggesting that *h*RyR3 is linked to Alzheimer’s disease [25,26]. Recently, the SARS-CoV2 spike protein was found to bind to *h*RyR2, producing a range of symptoms similar to those for ARVC/D2 [27]. Structural studies on mutant forms of RyR1 [13] and RyR2 [12] have described the structural effects of some of these mutations. In most cases, the mutation altered the closed state of the channel such that it either began to resemble the open form or adopted a half-open conformation, thereby reducing the stimulus needed to open it completely.

Malignant hyperthermia occurs upon the exposure of susceptible individuals to certain volatile anesthetics and is characterized by rigidity, acidosis, hypercarbia, and hyperthermia [28]. The mutations that cause malignant hyperthermia normally lead to increased Ca^2+^ release from the sarcoplasmic reticulum, which leads to increased Ca^2+^ binding to the myofilament, causing, in turn, dysregulated skeletal muscle contraction. This contraction provides the rigidity and the consequent hypermetabolism that causes the temperature rise that gives this disease its name. It is successfully treated using the muscle relaxant dantrolene, a derivative of hydantoin, which directly interacts with *h*RyR1 [29]. Recently, the likely dantrolene binding site in RyR1 was identified, and some of its effects on the structure of a disease-associated mutant were described [13].

**Figure 1:**
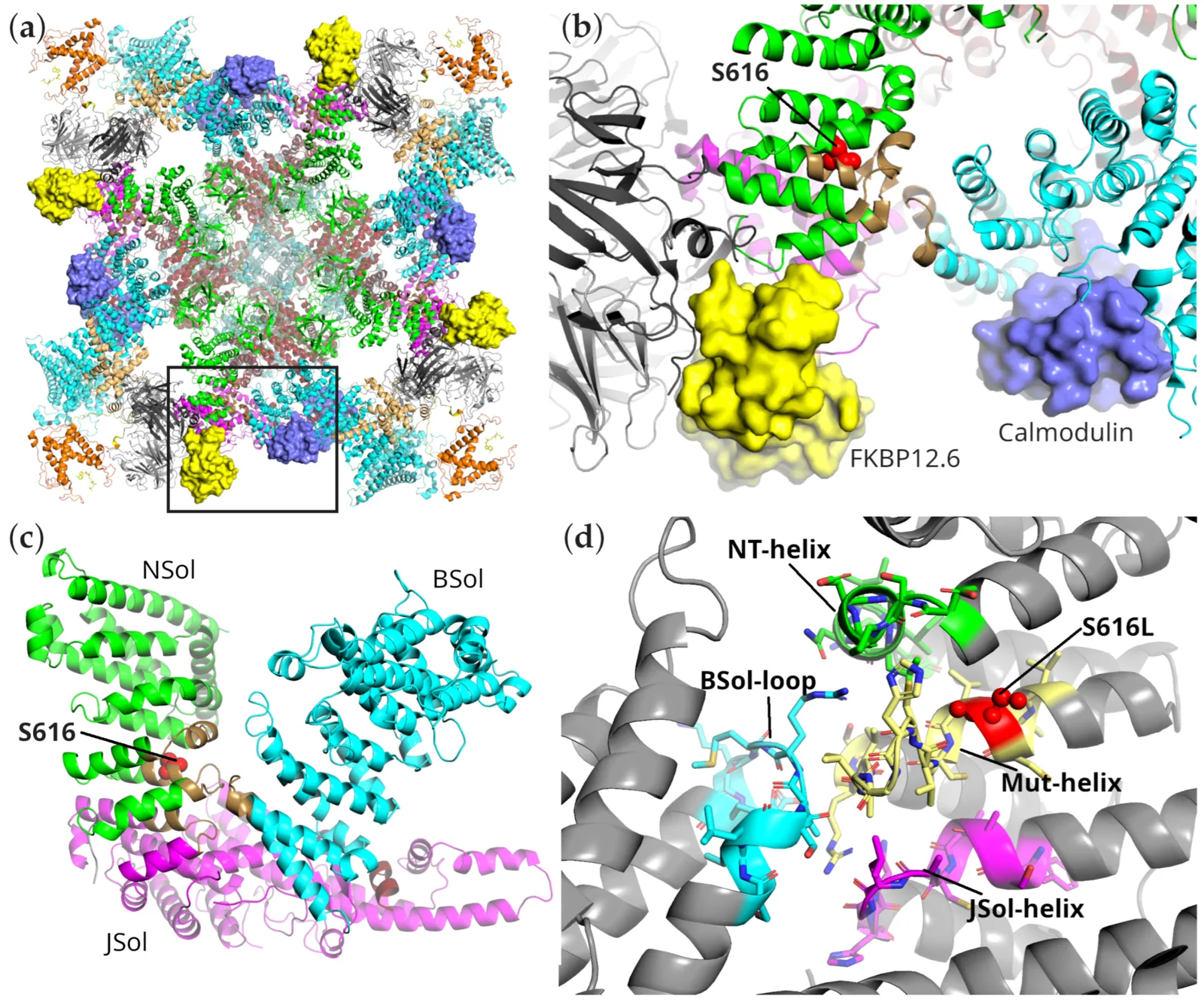
The *h*RyR2 tetramer. (**a**) The complete tetramer with a view down the channel from the cytoplasmic side. FKBP12.6 (yellow) and calmodulin (slate) are shown in surface representation bound to the protein. The *h*RyR2 domains are colored as follows: NTD, green; SPRY1, light gray; SPRY2, middle gray; SPRY3, dark gray; RY12, orange; JSol, magenta; BSol1 and BSol2, cyan; RY34, light orange; CSol, dark red; transmembrane Ion Channel domain, dark teal. (**b**) A close-up view of the boxed area showing the S616L mutation site (red spheres) from one chain. It can be seen that the site lies close to the junction (sand-colored) of the NTD, JSol, and BSol domains. FKBP12.6 and calmodulin are labeled. (**c**) The fragment used for MD simulation. All residues are from the cytoplasmic region. The NTD–JSol–BSol junction together with the S616L mutation site lies near the center of the fragment. (**d**) The fragment in (**c**) after rotation by 180° about the vertical axis and zoomed in on the three-domain interface. Here the individual residues that comprise the interface are highlighted and colored as follows: from the NTD, there are the NT-helix (green, residues 581–591) and the Mut-helix (yellow, residues 613–627); from the JSol domain, there is the JSol-helix (magenta, residues 1661–1670); and from the BSol1 domain, there is the BSol-loop (cyan, residues 2128–2138). The structure shown in (**a**,**b**) is 7u9t and that in (**c**,**d**) is 7ua5, both from Miotto et al. (2022) [12].

Similarly, CPVT1, a ventricular tachycardia triggered by stress, is also based on excessive Ca^2+^ leakage from the sarcoplasmic reticulum in cardiomyocytes [30,31,32]. A large number of mutations to the RYR2 gene are associated with CPVT1 [22,23,32,33,34,35]. One CPVT-associated mutation, S616L, was identified in a female adolescent who exhibited exercise-induced syncope [33]. The residue affected occurs in the next-to-last α-helix of the N-terminal domain, close to the junction of three different, sequentially non-adjacent domains in *h*RyR2 (Figure 1): the α-helix containing the mutation (residues 607–618) forms part of the junction between the N-terminal domain and one of the first α-helices of the JSol domain (residues 1648–1665); Ser-616 itself forms part of the C-terminal end of this helix, and the residues following it (617–624) also form part of a loop that interacts with residues 2131–2136 of the BSol domain. This area is also adjacent to the FKBP12 binding site and near a part of the Bsol domain, which is affected by calmodulin binding. Calmodulin is known to inhibit RyR2 [36], while FKBP12.6 (also known calstabin-2) may act to stabilize the closed form of RyR2, though there is substantial uncertainty as to what its role actually is [37]. To the best of our knowledge, the effects of this mutation have not been otherwise studied.

Both malignant hyperthermia and CPVT1 have been suggested to arise from Ca^2+^ leakage from defective RyR channels as a consequence of domain “unzipping” [38,39]. Whether or not this hypothesis is correct generally, the location of the S616L mutation suggests that it may be the case here. To see how this domain junction might become “unzipped”, we carried out a molecular dynamics simulation on a fragment containing the mutation site and the area adjacent to it and compared this to a simulation containing the S616L mutant. We also explored the effect of this mutation at the molecular level by cloning, expressing, purifying, and characterizing a 655-residue fragment containing the complete *h*RyR2 NTD in both wild-type and S616L-containing forms and evaluated the change in thermal stability of this fragment caused by the mutation.

## 2. Results

### 2.1. Molecular Dynamics Simulation

Point mutations within the ryanodine receptor can have unexpected effects at relatively distant areas of the channel [12,13,40]. For this reason, the best approach to studying the effects of these mutations would be to simulate the entire channel. However, the channel is extremely large, with each chain containing nearly 79,000 atoms and the four chains that comprise the complete receptor having nearly 316,000 atoms in total. Moreover, the dynamics of the channel operate on long timescales where the microsecond time-frame of and all-atom molecular dynamics simulation is typically not long enough to observe channel gating. To avoid these issues, previous MD studies on the pore dynamics of RyR1 [41,42,43] and RyR2 [44] have examined smaller fragments containing only the channel domain and part of the central (CSol) domain. In light of this background, we chose to address a more modest question: what would be the likely effect of the S616L mutation on the structure of the immediately surrounding area? To answer this question, we created a structure containing residues 409–647, 1641–1763, 1777–1854, 1889–2010, 2053–2364, 2377–2444, and 2455–2476. These residues create a region containing all of the α-helical solenoid of the NTD (NSol), nearly all of the JSol domain, and almost 2/3 of the BSol1 domain (Figure 1c, Table 1). Several surface loops in the JSol and BSol1 domains were either truncated or left unmodeled to simplify the analysis; their broken ends were fused together to create a continuous chain. BSol1 was truncated based on a normal-mode analysis, which suggested that residues 2111–2476 (ca.) moved as a collective unit. The final constructed fragment contained only around 15,480 atoms, within the limits of our resources to simulate and, moreover, contained only residues from the cytosolic region that do not lie near the membrane zone, thus allowing us to carry out our simulations without including a lipid bilayer.

This fragment contains the entirety of the three-way NSol-JSol-BSol junction. The residues that most contribute to this interface are 581–591 (henceforth the NT-helix) and 613–627 (Mut-helix) from the NTD, 1661–1670 (JSol-helix) from the JSol domain, and 2128–2138 (BSol-loop) from the BSol1 domain (Figure 1d, Table 1). Although there are additional residues involved in the interfaces between the NSol and JSol domains and between the JSol and BSol domains, only the residues in these four loops take part in the interface between all three domains, and these will be the main focus of the analyses described below. The closed form of the protein was used as the base conformation because it is this form that is expected to be the most disturbed.

There is evidence that *h*RyR2 phosphorylation is necessary for the induction of CPVT1 in at least some mutations [45]. Unfortunately, the conformation of the fragment used in the present study is almost identical to that of a similar fragment taken from the PKA-phosphorylated *h*RyR2 structure reported by Miotto et al. (2022) [12]. Consequently, the influence of PKA-phosphorylation on the mutation site and the influence of the S616L mutation on phosphorylated *h*RyR2 cannot be addressed in this study.

Six simulations were done for each of the wild-type and S616L mutant systems using different random seeds for the initial velocities. First, a 150 ns simulation was performed. Inspection of the RMSD matrix and the potential energy plot for these simulations showed that both forms had equilibrated by 30 ns. Four 70 ns simulations were then run for each system to give six independent trajectories for each one. The last 120 ns from the first simulation was then merged with the final 68 ns of each of the second four simulations to give a 460 ns merged trajectory for each system. These merged trajectories were subsequently analyzed and compared. To facilitate comparisons between the wild-type and mutant trajectories, a joint trajectory was created by fusing two trajectories containing only the Cα atoms from the wild-type and mutant trajectories.

#### 2.1.1. Initial WT Trajectory Analysis

Before comparing the wild-type and mutant trajectories, it is necessary to ensure that the wild-type behavior occurs consistently given the short trajectories used. The properties we are most interested in here derive from the conformational distribution of the trajectories, so the root-mean-squared deviations of the residues in the interface loops should be examined. Figure S1 in the Supplementary Material shows that the time courses of the trajectory RMSD with respect to the starting structures generally remain within a relatively narrow band over the course of the simulations. There is also no gradual increase or decrease observed, meaning that the interface in the wild-type simulations remains relatively stable. Figure S2 compares the mean RMSDs of the simulations and shows that all but one of them are broadly similar. Grouping the structures using a clustering analysis with a 1.0 Å cutoff (Figure S3) shows that there are two main conformations, one of which is clearly preferred, being the overwhelmingly dominant form in four of the six trajectories and the preferred one in the fifth. Only one of the trajectories clearly preferred the alternative trajectory. This is also reflected in the center-of-mass distance plots (Figure S4), which likewise show very similar average values over four of the six trajectories with only one trajectory being markedly different (the fifth trajectory, which is split between the two forms, has a value midway between them). The conformational state of the wild type under these conditions is therefore consistent enough to proceed with a comparison to the mutant.

#### 2.1.2. RMSD and Clustering Analyses

**Figure A1:**
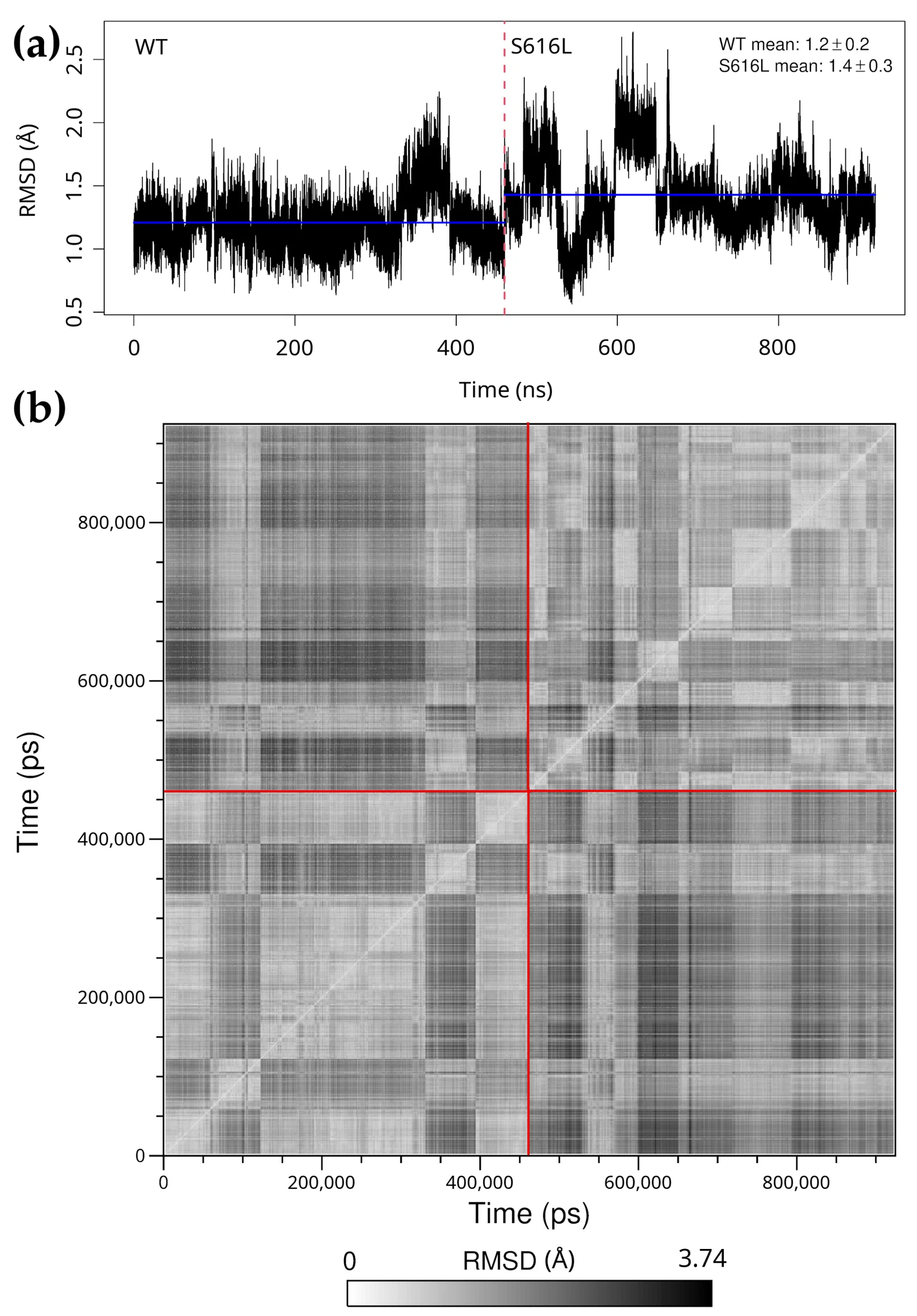
The root-mean-squared deviation (RMSD) of a joint trajectory containing frames from both the wild-type (0–460 ns) and mutant (461–920 ns) trajectories. (**a**) A one-dimensional RMSD plot showing the RMSD of the joint trajectory relative to the wild-type starting structure. The two halves of the trajectory are separated by the dashed red line and are labeled in the upper corners. The solid blue lines indicate the average value of the wild-type and mutant RMSDs. The mutant RMSD is both larger and more heterogeneous. (**b**) The RMSD matrix of the same trajectory showing the RMSD of every structure in the trajectory with every other structure. The division between the wild-type and mutant trajectories is marked by crossed red lines. It is also apparent that the two principal conformational states in the wild-type half of the matrix also occur in the mutant form, though with diminished probability.

To verify that the interface does not greatly change over the course of the simulation, we calculated the Cα RMSD of the interface loops described above with respect to the starting structure. Figure A1a shows that the wild-type structure has an average RMSD of 1.2 ± 0.2 Å relative to the initial structure and that this RMSD remains within a relatively narrow band over the course of the conjoined trajectory, indicating that the interface in the wild-type simulation remains relatively unchanged. By way of contrast, the mutant trajectory has a higher average RMSD (1.4 ± 0.3 Å) and also shows several clear variations, which correspond to some of the different simulation blocks. Despite this, the mutant also has a relatively stable region, which likely corresponds to a separate, stable conformation.

Examining the RMSD matrix of the joint trajectory (Figure A1b), which shows the RMSD of every structure in the trajectory with every other structure, shows that the wild-type trajectory is relatively conformationally homogenous, with only two main conformational states. The mutant trajectory, on the other hand, is more heterogeneous with 4–5 main conformational states.

**Figure 2:**
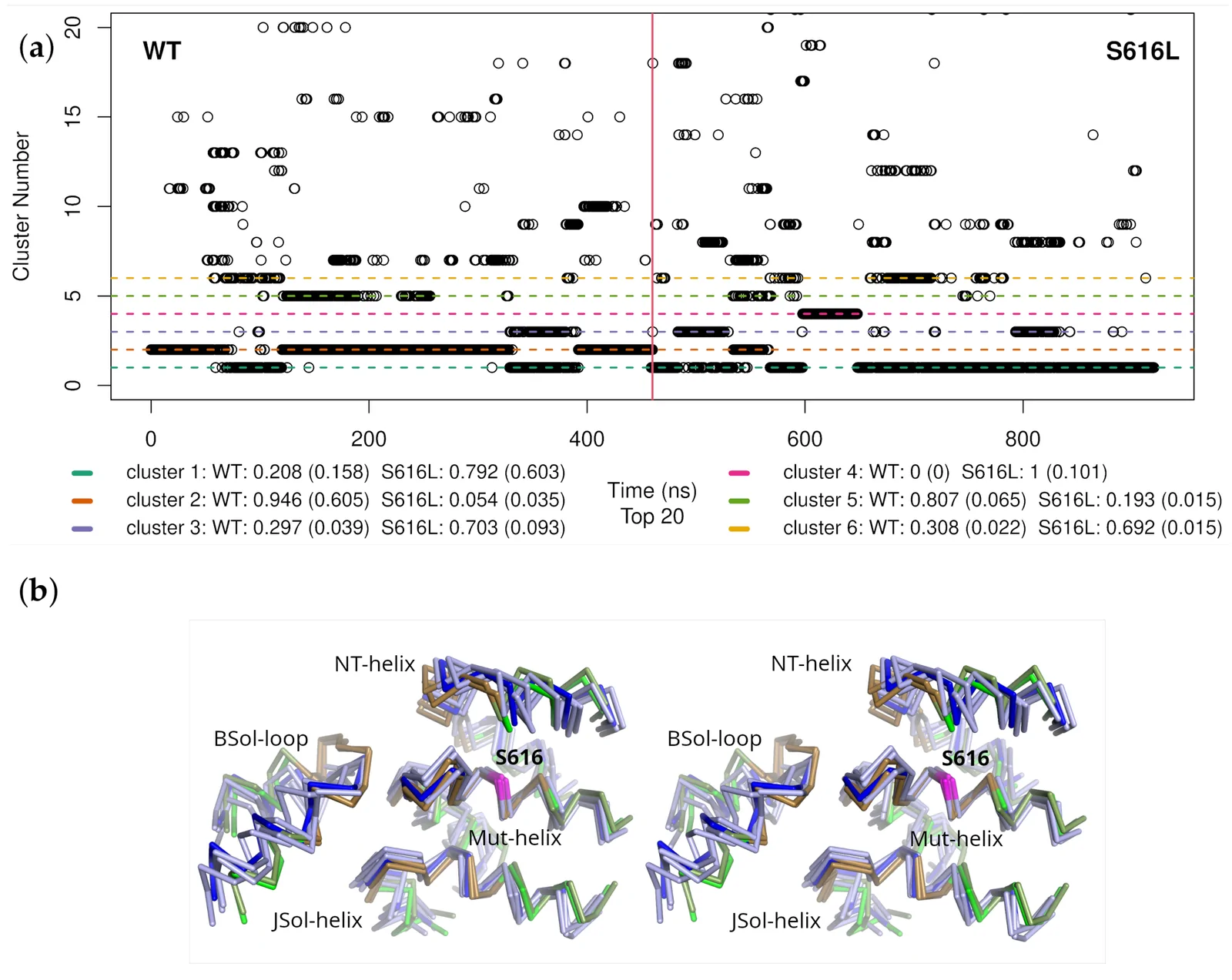
Cluster structures. (**a**) A plot of trajectory time-stamp versus cluster number (1.0 Å cutoff). The largest clusters containing the most frames are at the bottom. The red line at 460 ns marks the division between the wild-type half of the trajectory and the S616L mutant half. It can be clearly seen that there is a qualitative difference between the wild-type and mutant halves. The six largest clusters are marked out using differently colored dashed lines. For each marked cluster, the legend below gives the fraction of the cluster contained by the wild type (WT) and S616L mutant (S616L) together with the fraction of the entire wild-type or mutant trajectory described by this cluster (thus, the wild type has 20.0% of cluster 1, and cluster 1 contains 15.8% of all wild-type frames, while the mutant has 79.2% of cluster 1, and cluster 1 contains 60.3% of all mutant frames). Representative Cα traces of the first six clusters are shown in (**b**). (**b**) is a stereo view that compares the two majority wild-type clusters (2 and 5, green and sandy brown) to the main mutant cluster (1, blue) and the top majority mutant clusters (3, 4, and 6, light blue). The wild-type clusters are colored green, and the interface residues, colored sandy brown, are explicitly labeled. Residue 616 is colored magenta in (**b**).

The conformational differences can be more clearly seen in Figure 2, where a clustering analysis of the joint RMSD matrix has been carried out using an RMSD cutoff of 1.0 Å. The top six clusters, which contain nearly 90% of the structures in both the wild-type and mutant trajectories considered separately, are highlighted. This figure shows that the wild type and mutant each have one major cluster that contains 60% of their conformations. The principal WT cluster is cluster 2 (the wild type contains 95% of cluster 2, and cluster 2 comprises 61% of all wild-type conformations), followed distantly by cluster 5 (the wild type has 81% of all the structures in cluster 5, but cluster 5 comprises only about 6.5% of the wild-type conformations). The dominant cluster of the S616L mutant is cluster 1, which contains 60% of all mutant conformations, and the mutant has 79% of all cluster 1 conformations. The mutant is the main contributor to clusters 3 and 6 and is the only contributor to cluster 4, but these clusters comprise only 9.3%, 1.5%, and 10%, respectively, of the total mutant conformations. Overall, the WT has a total of 27 clusters, while the mutant has a total of 29. They share clusters 1, 2, 3, 5, 6, 7, 8, 9, 11, 12, 13, 14, 16, 18, 20, 24, 25, 27, and 34 (i.e., 19 of these are shared, so the WT has 8 unique and the mutant has 10 unique). Since the top 6 contain about 90% of the conformations of both the WT and mutant, most of the unique ones contribute very little.

**Figure A2:**
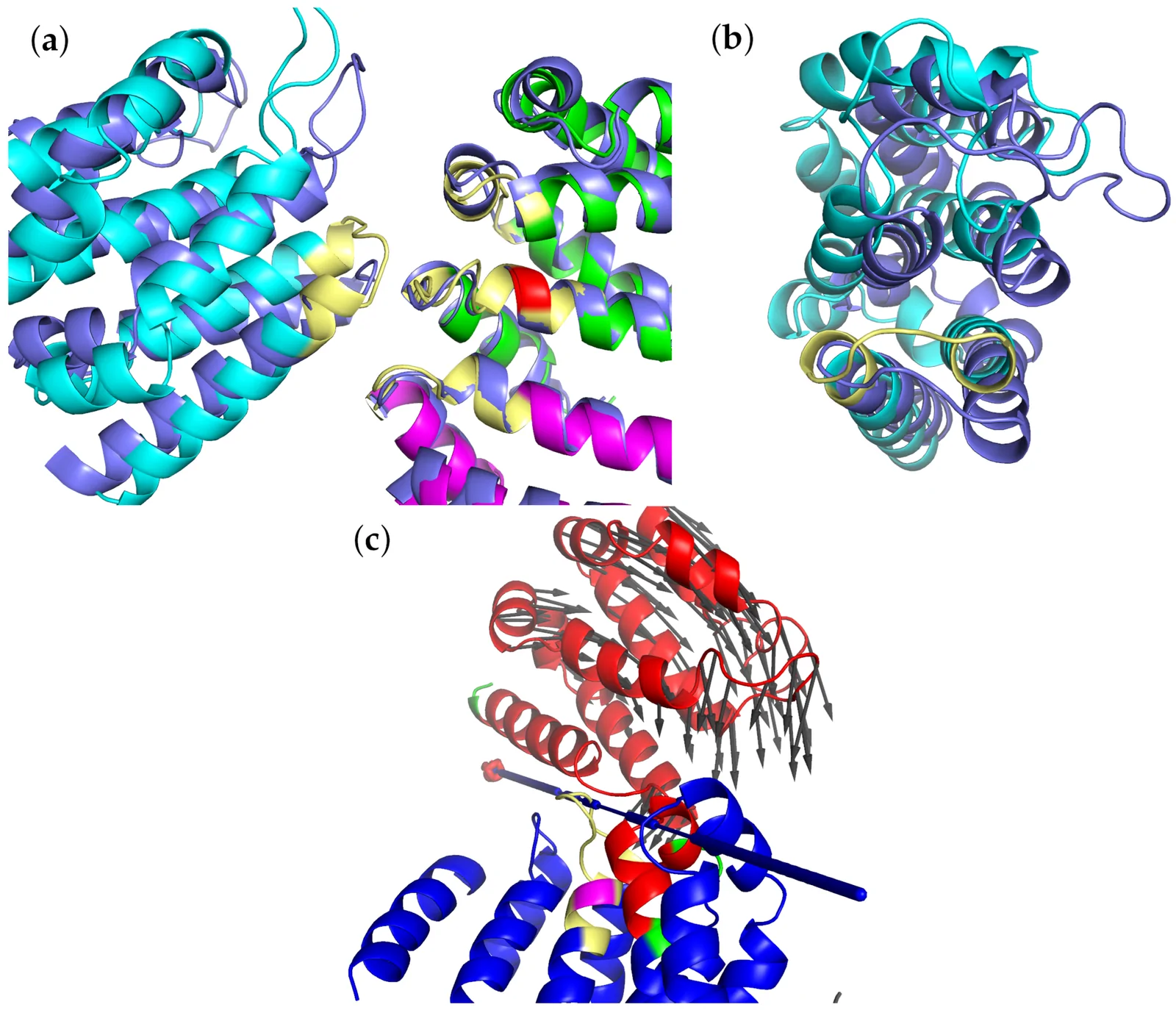
The central structures of clusters 1 and 2 from the clustering analysis. Cluster 2, which corresponds to the predominant wild-type fragment, is colored by domain as in Figure 1b but with the interface loops pale yellow. Cluster 1, which corresponds to the main mutant fragment, is colored slate. (**a**) A view of the interface showing the shift of the BSol domain in the mutant trajectory. (**b**) A view roughly 90° from (**a**) to more clearly show the rotation of BSol. (**c**) An analysis of the shift between these two structures according to DynDom [46]. The stationary fragment containing the NSol and JSol domains is blue, while the moving BSol fragment is red. The blue stick corresponds to the axis of rotation, and the arrows show the motion of the BSol fragment from the wild-type conformation to the mutant one.

The central structure of each cluster, the one with the smallest RMSD from all other structures of the cluster, is illustrated in Figure 2b. All six structures show that the JSol loop has changed very little, the mutation loop has changed slightly more (the range of motion for the loop immediately following the end of the α-helix seems to have increased slightly in the mutant trajectory), and the NT-helix appears to have moved noticeably away from the BSol domain. The BSol-loop shows by far the greatest change. In the clusters that contain only or mostly conformations from the mutant trajectory (1, 3, 4, and 6), the BSol-loop has undergone a clockwise rotation of about 18.6° with respect to the NSol domain together with a 1.5 Å translation that brings its C-terminal half, residues 2133 and following, away from the NT-helix and towards residues 624–627 at the C-terminal end of the Mut-helix loop (the rotation and translation is from DynDom [46]; see also Figure A2). This also has the effect of changing the disposition of the BSol-loop with respect to the C-terminal residues of the JSol-helix. Although the general motion of the BSol-loop brings it closer to the JSol-helix, it does tend to move the N-terminal residues of the BSol-loop away from those in the loop at the C-terminal end of the JSol-helix.

Overall, the mutation has changed the preferred conformation of the interface residues, changing the preferred conformation from that represented by cluster 2 to that represented by cluster 1. Although four of the top six clusters, and in particular the two largest ones, are shared by both the wild-type and mutant forms, the mutation has greatly changed their relative proportions. Furthermore, although the mutant form does have conformations that are not shared with the wild type, all the wild-type conformations are also accessible by the mutant. Thus, the mutation has the effect of disfavoring the most common wild-type conformation while promoting the formation of other, otherwise disfavored conformations.

#### 2.1.3. Distances Between Interface Loops

**Figure A3:**
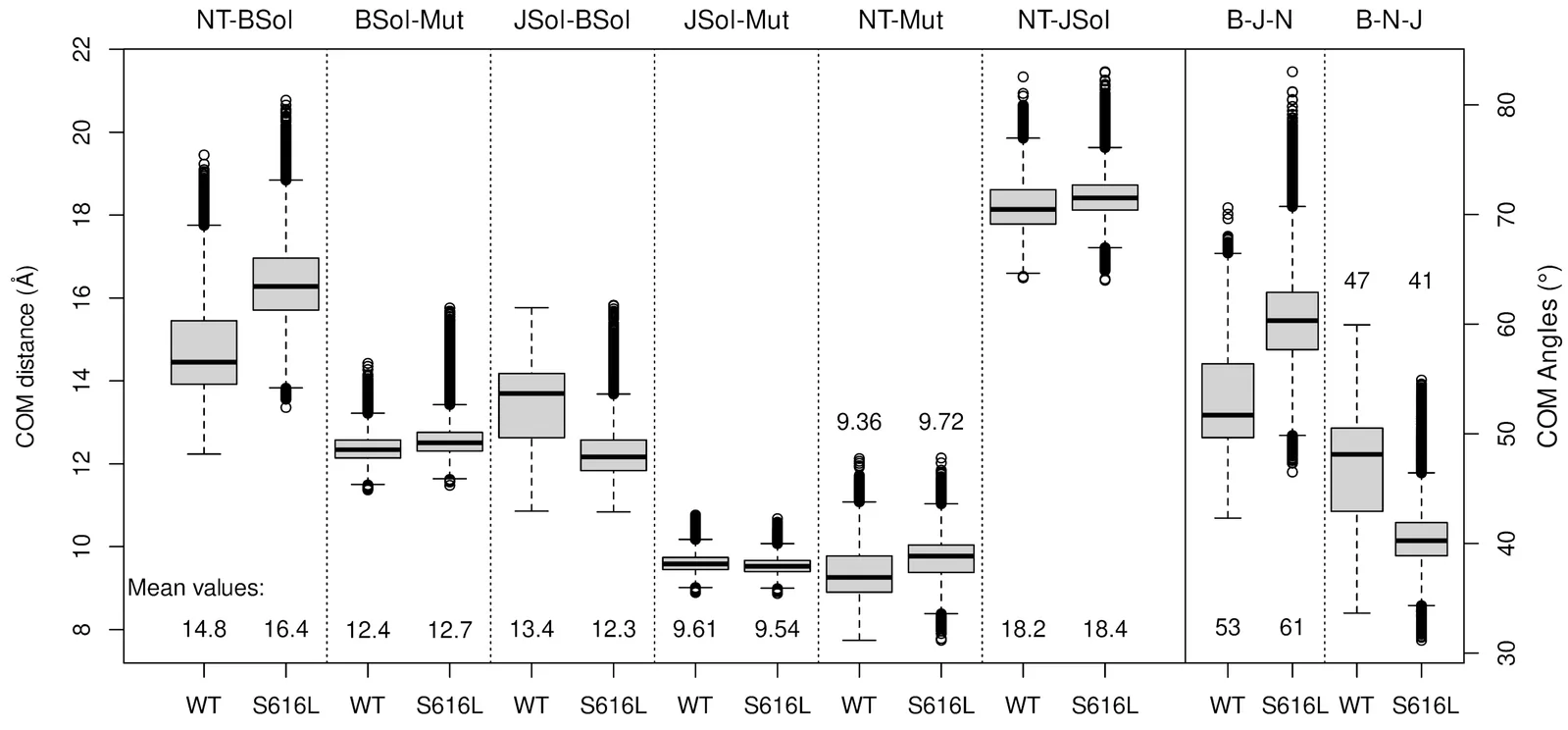
Boxplots showing the distances between the centers of mass of the Cα atoms of the individual interface loops in both the wild-type and S616L mutant structures. The left-hand side gives the COM distances in Å; the right-hand side gives the size of the angles between three of the loops in degrees. The mean values for each entry are printed below or above the individual boxplots. The loops are abbreviated as follows: NTD—NT-helix, BSol—BSol-loop, Mut—Mut-helix, JSol—JSol-helix; B-J-N—angle between the BSol-loop, JSol-helix, and NT-helix; B-N-J—angle between the BSol-loop, NT-helix, and JSol-helix.

To more throughly quantify the changes to the interface, we also calculated the differences in the distances between the centers-of-mass (COM) of the Cα atoms of the interface loops. Boxplots summarizing the results can be seen in Figure A3. The measurements showed that the BSol-loop–NT-helix distance increased by about 2 Å and the BSol-loop–Mut-helix distance increased by about 0.3 Å as a result of the mutation, while the BSol-loop–JSol-helix distance decreased by about 1 Å. The S616L mutation also caused the corresponding BSol-JSol-NT angle to increase by about 8°, while the BSol-NT-JSol angle decreased by about 7°. This shows that the BSol-loop is moving away from the NSol domain (which contains both the NT-helix and the Mut-helix) but towards the JSol domain.

#### 2.1.4. Root-Mean-Squared Fluctuations

**Figure 3:**
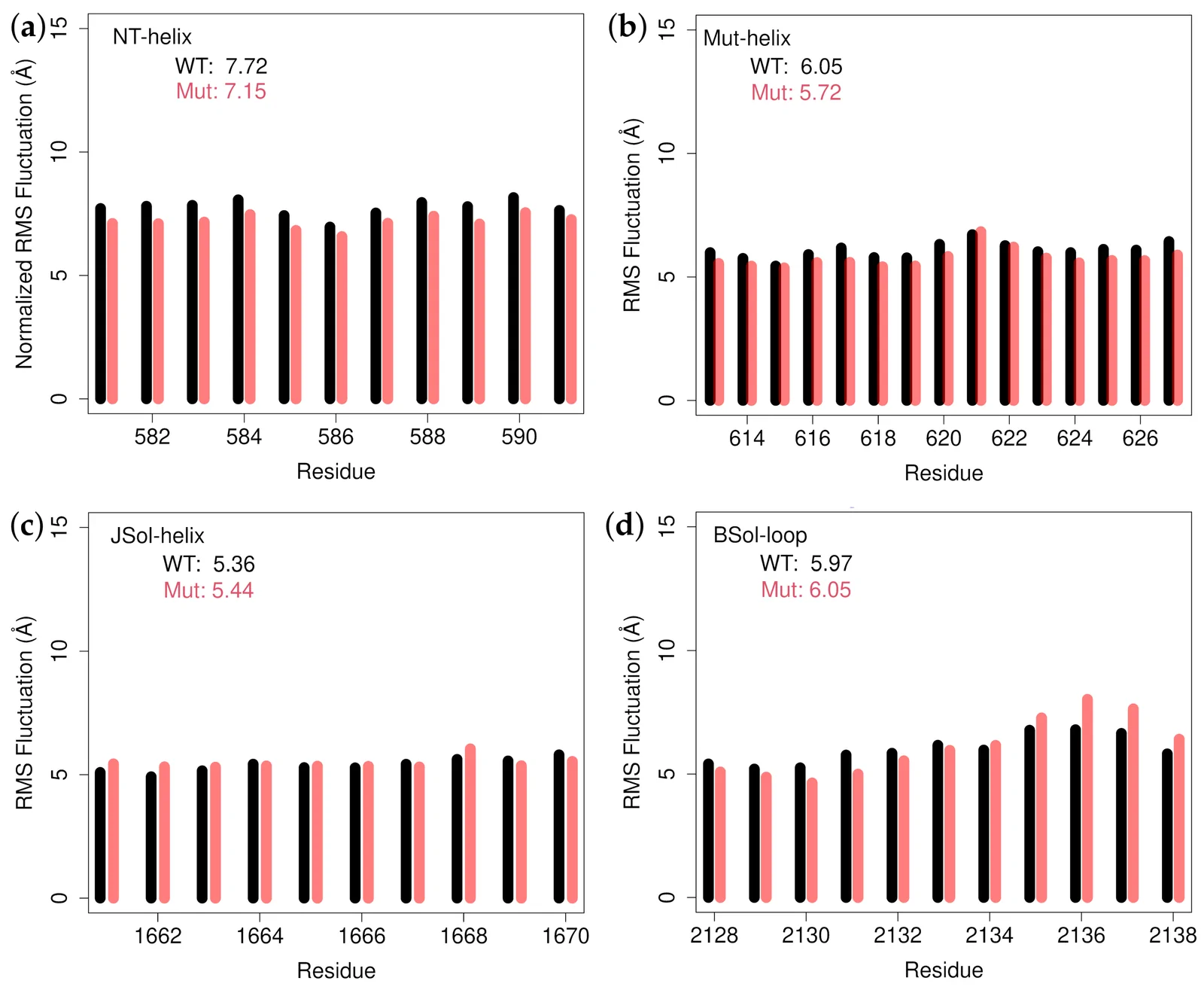
Differences in the root-mean-squared fluctuations (RMSFs) between the Cα atoms of the wild-type and mutant proteins in (**a**) the NT-helix, (**b**) the Mut-helix, (**c**) the JSol-helix, and (**d**) the BSol-loop. In each panel, the wild-type RMSF for each residue is represented by the black bar, while the red bar represents the RMSF of the S616L mutant. The numbers in each panel indicate the mean RMSF of the residues in each panel.

**Figure A4:**
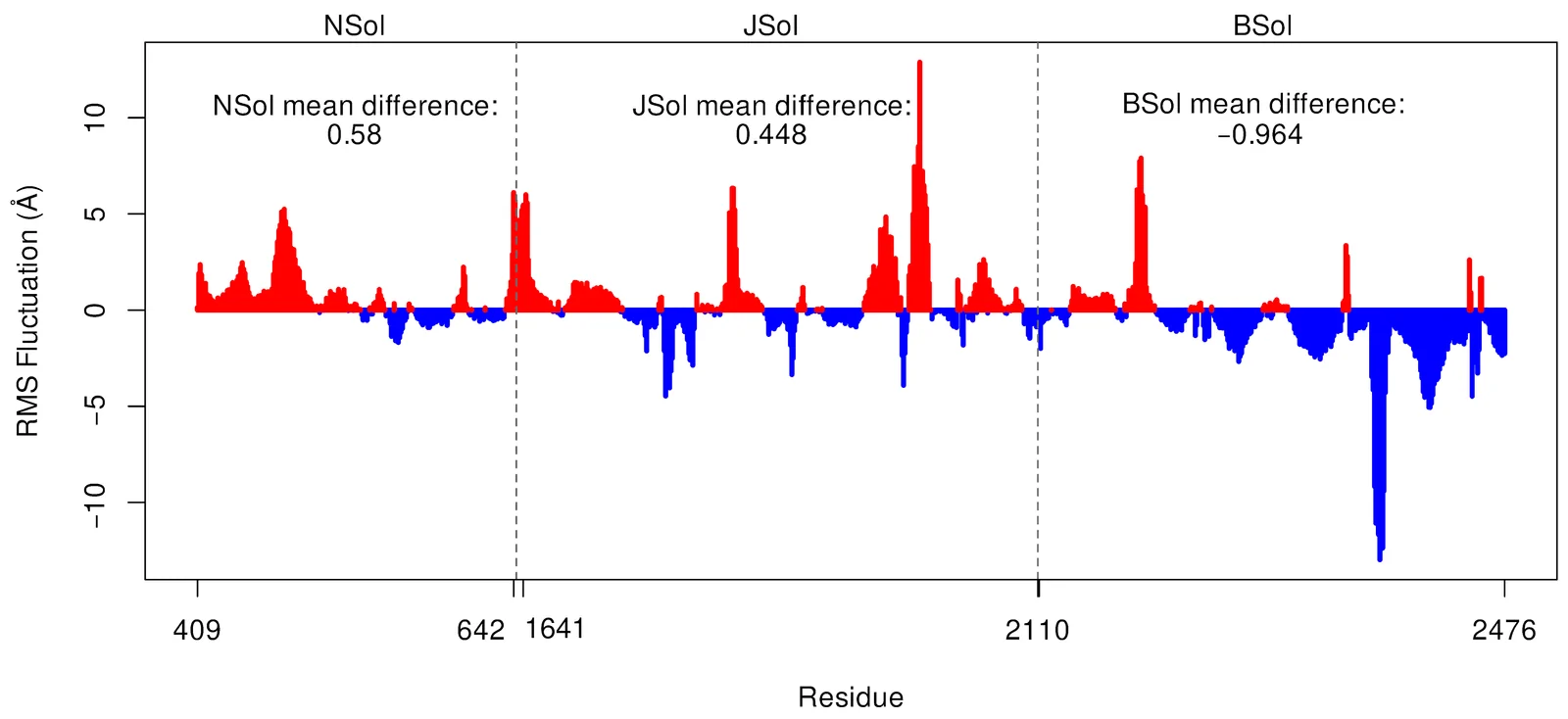
A plot showing the differences in normalized root-mean-square-fluctuation (RMSF) of the Cα atoms between the sets of structures containing the preferred wild-type and mutant conformations (that is, between the populations of wild-type cluster 2 and mutant cluster 1). Red areas represent areas where the mutation has increased the overall RMSF; blue areas show locations where the mutation decreased the overall RMSF. The mean differences are given at the top of the plot for each domain, and the boundaries of the individual domains have been marked with dashed lines and are labeled at the top of the plot. To more clearly show how the individual areas increased or decreased relative to the other parts of the structure, the RMSFs of each trajectory were normalized by their corresponding average values.

One of the questions worth addressing is the difference in dynamic motion between the preferred wild-type conformation and the preferred mutant one. Although the dynamics of the ryanodine receptor cannot be adequately sampled by the relatively short simulations described here, some insight into the dynamic motion can be gained by comparing the root-mean-squared fluctuations (RMSFs) of the main clusters of the wild-type and mutant trajectories (clusters 2 and 1 in Figure 2 above). To explicitly follow only the motion of the protein backbone, the RMSFs of only the Cα atoms will be considered. To properly analyze how the mutation has changed the relative degree of mobility of each part of the fragment relative to the whole, the RMSF needs to be normalized by dividing by the average value of the corresponding trajectory. Figure A4 shows the differences in normalized RMSF between the wild-type and mutant forms for the entire fragment. It can be seen that the overall fluctuations of the NSol and JSol domains has increased by about 0.58 and 0.45 Å, respectively, while the fluctuations the BSol domain has decreased by nearly 1 Å (0.96 Å). Examining the interface residues themselves (Figure 3) shows that the preferred mutant conformation has decreased the average RMSF of the two loops belonging to the NSol domain, the NT-helix, and the Mut-helix while slightly increasing the average RMSF of the JSol-helix and BSol-loop.

Looking more closely at the individual residues in Figure 3, we see that all residues in the NT-helix and Mut-helix aside from His-621 have slightly decreased in flexibility. The JSol-helix shows an overall increase in motion outside of three of the final four C-terminal residues, which are the ones closest to the BSol-loop. The BSol-loop shows a clear split between its C-terminal and N-terminal ends. The six N-terminal BSol-loop residues all show decreased RMSF; these are the residues that are opposite to and interact with the JSol-helix and the loop following the Mut-helix. The five C-terminal BSol-loop residues, which are opposite to and would interact with the NT-helix, all show an increase in RMSF, which does not completely counterbalance the decrease in the N-terminal residues. That is, the N-terminal residues of BSol, which are in contact with the JSol domain, are stabilized, while the C-terminal residues, which are closer to the NT-helix in the wild-type conformation, become destabilized. This suggests that the mutation in the NSol domain has encouraged a conformational change that disrupts the BSol-loop side of the three-way interface by stabilizing it via a different conformation.

#### 2.1.5. Principal Components Analysis

**Figure A5:**
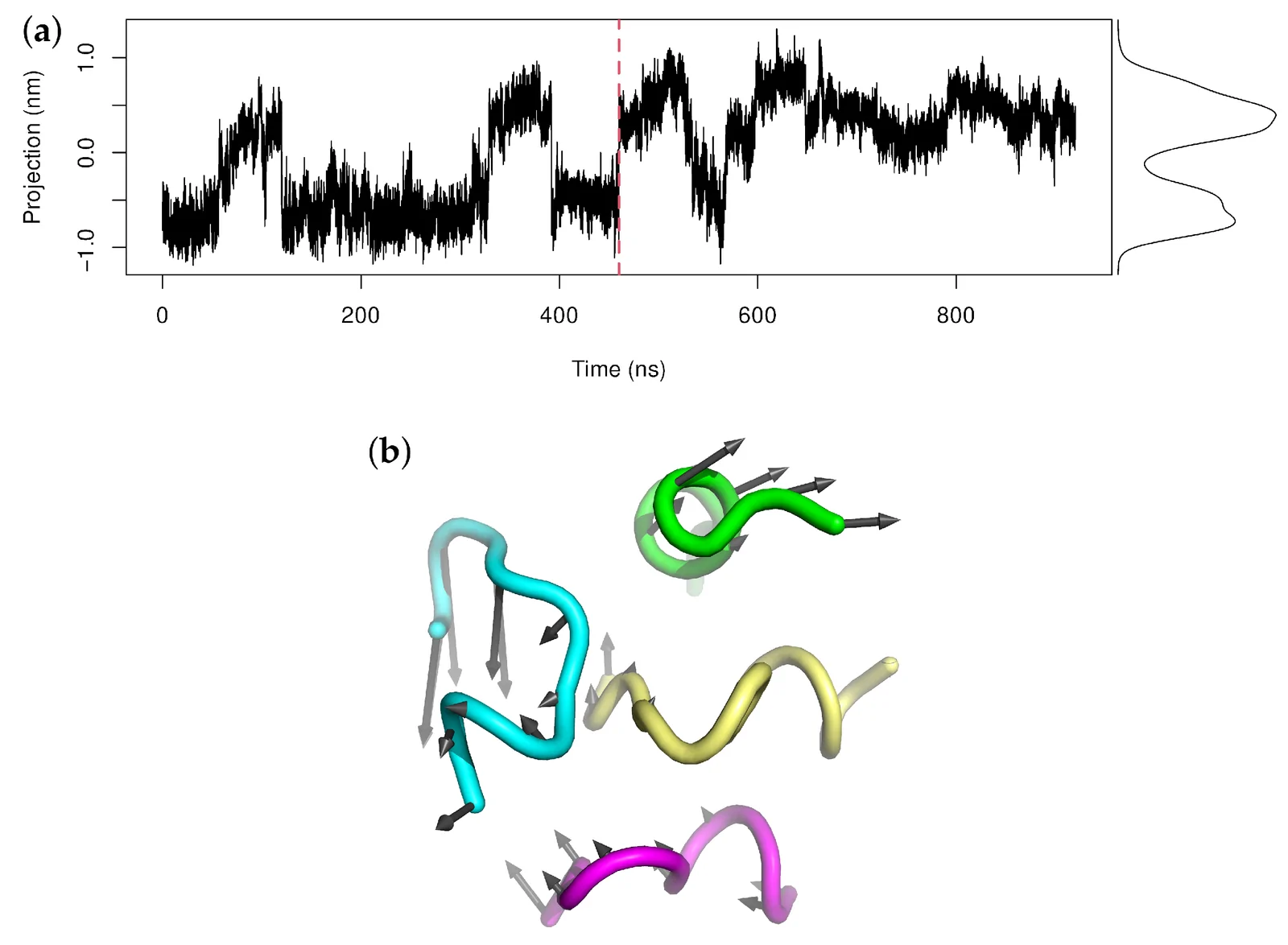
The first principal component of the joint matrix shows the vector that describes the greatest difference between the wild-type and mutant trajectories. This is very similar to PC 1 of the individual wild-type and mutant trajectories, which describes the transition between the structures represented by clusters 1 and 2. (**a**) The projection of the first principal component on the joint trajectory. The red dashed line marks the division between the wild-type (left) and mutant (right) halves. (**b**) A Cα trace showing the extreme positions of this principal component.

An examination of Figure A1 shows that the difference between the wild-type and mutant halves of the joint trajectory can be easily distinguished. Therefore the first principal component (PC) should describe the main difference between the wild-type and mutant trajectories. Figure A5 shows the one-dimensional projection of the first PC on the joint wild-type–mutant trajectory together with an illustration of the extremes of the motion. It can be seen that the transition from the wild-type to the mutant form involves large motions of the NT-helix and the C-terminal half of the BSol-loop away from each other together with smaller motions of the C-terminal end of the JSol-helix and the N-terminal end of the BSol-loop towards each other. Comparing Figure A5b to Figure 2b shows that this vector largely describes the motion that would convert the conformation described by cluster 2, the preferred wild-type form, to cluster 1, the preferred mutant form.

**Figure A6:**
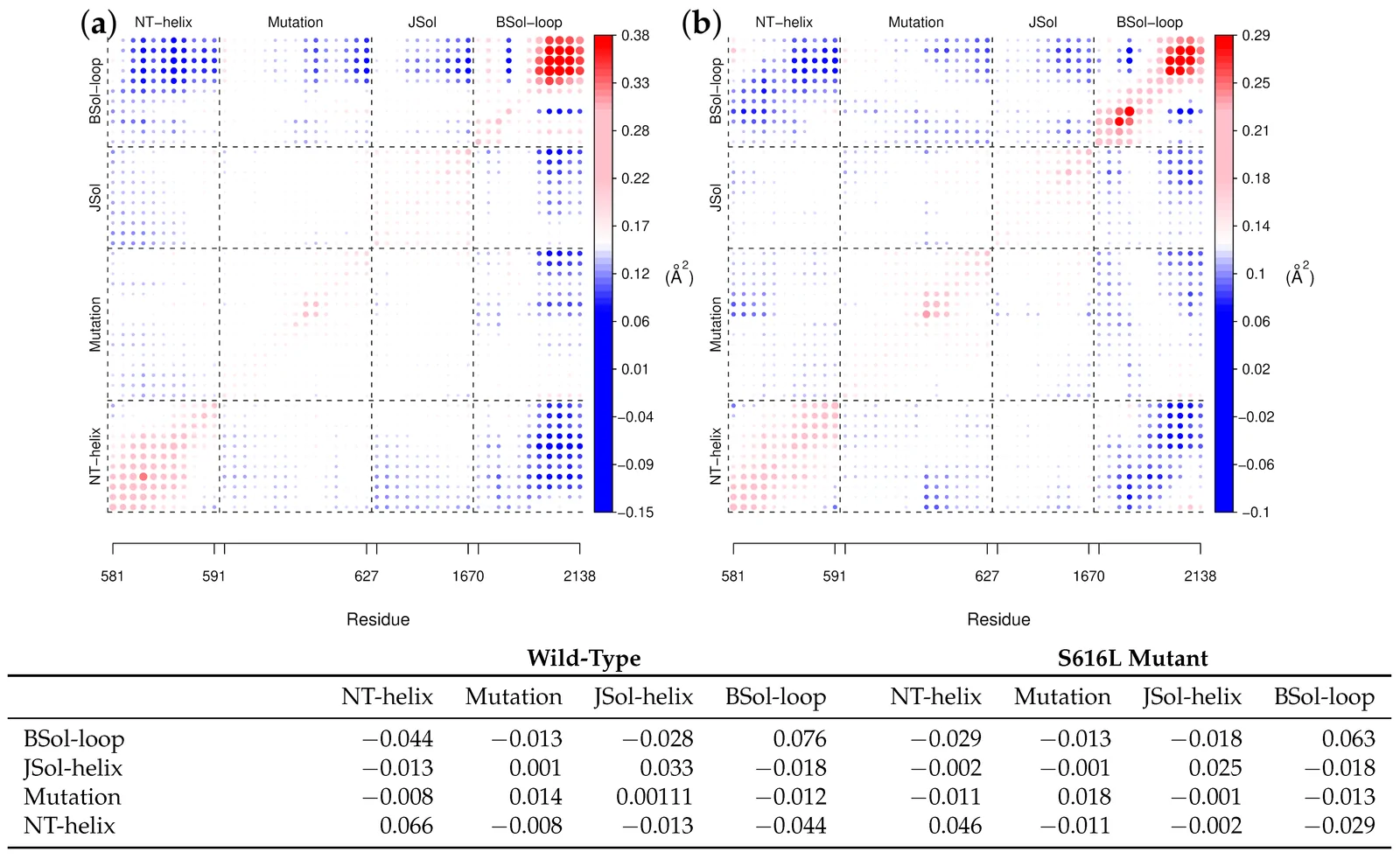
The averaged covariance matrices of the wild-type (**a**) and mutant (**b**) trajectories calculated over the Cα atoms. The gray lines mark off the regions corresponding to the given flexible regions. To aid in interpretation, the table at the bottom shows the average covariance of each region.

**Figure A7:**
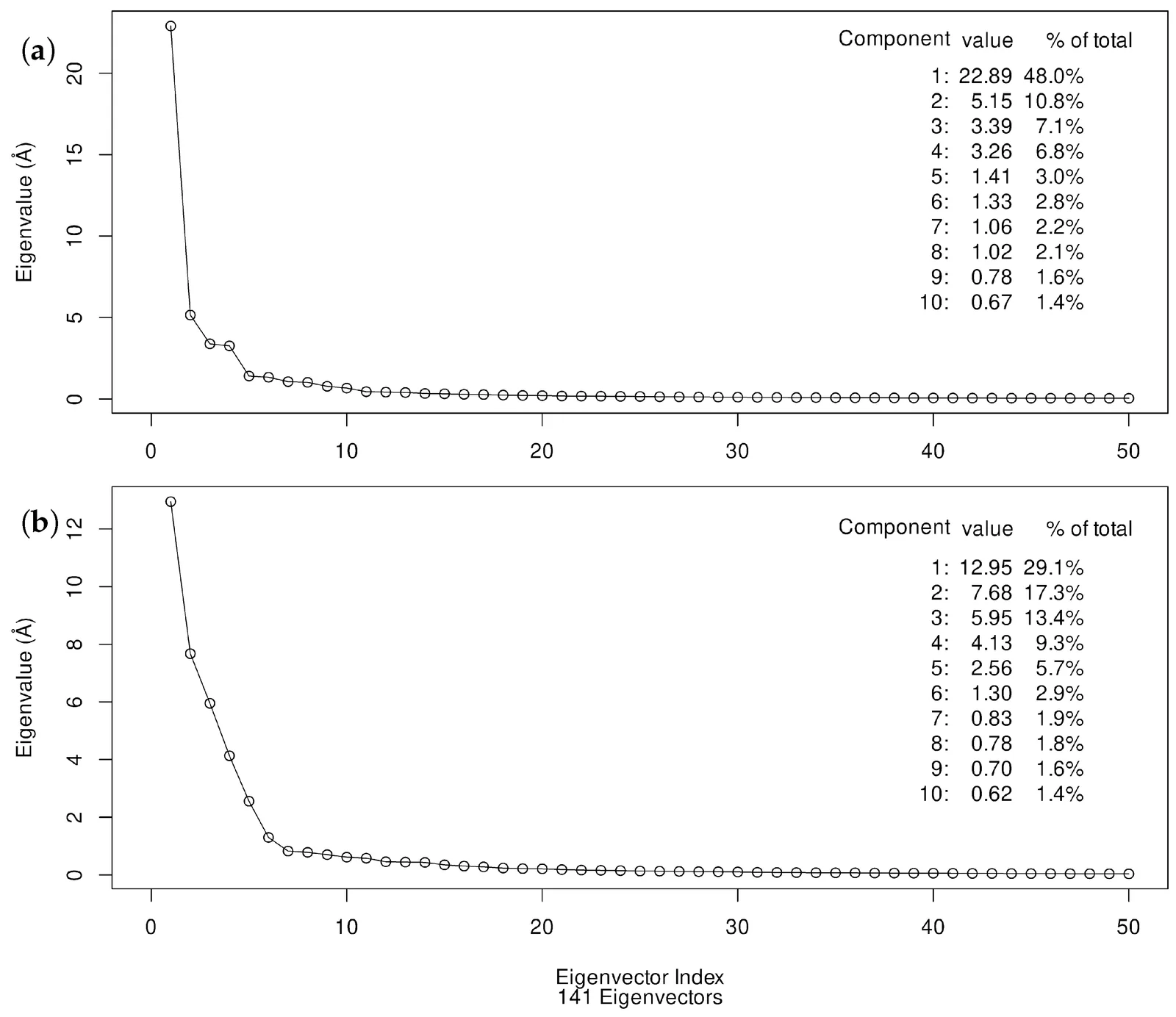
The eigenvalue scree plots for the wild-type (**a**) and S616L mutant (**b**) trajectories. Both plots show the first 50 of 141 total eigenvalues. Each plot has a table inset, which shows the magnitude and contribution to the total variance of the first 10 eigenvalues. The first 10 describe 85.8% of the total motion of the wild type and 84.4% of the total motion of the mutant. The top 5 capture 75.7% and 74.8%, respectively.

Principal components analysis (PCA), also called essential dynamics, is commonly used to identify the major modes of motion present in a molecular dynamics trajectory [47,48]. Since the wild type and S616L mutant can generally access the same conformations but with different preferences, we might expect them to generally have the same principal components of motion, but with different relative rankings. We carried out the analysis on only the positions of the Cα atoms of each protein, and only the residues in the four interface loops defined above were considered. The average covariance matrices are shown in Figure A6, and the eigenvalue scree plot is shown in Figure A7.

It can immediately be seen that, although the two covariance matrices do differ somewhat in their details, they are largely similar. Among the differences is that the diagonal regions corresponding to each of the four loops are slightly more clearly defined in the mutant matrix and that the N-terminal and C-terminal halves of the region corresponding to the BSol-loop are also more clearly distinguished from one another in the mutant matrix. Both forms show that the BSol-loop shows the greatest degree of variation with respect to the other three loops, but the mutant matrix shows a greater variety of interactions. Unfortunately, it can also be seen that the diagonal elements of the matrix are more than 3× greater than the off-diagonal elements in both matrices, meaning that describing the dynamics of the system will require several PCs. As shown in Figure A7, the first three PCs describe only about 66% of the dynamics of the wild-type protein and about 60% of the mutant. In both systems, five components are needed to describe approximately 75–80% of the motion, and ten are needed to describe about 90%. Examining the first five eigenvalues also shows that the mutation has reduced their relative importance. While the first five wild-type PCs describe 48%, 11%, 7%, 7%, and 3% of the motion, the top five mutant PCs describe 29%, 17%, 13%, 9%, and 6% of the motion.

**Figure A8:**
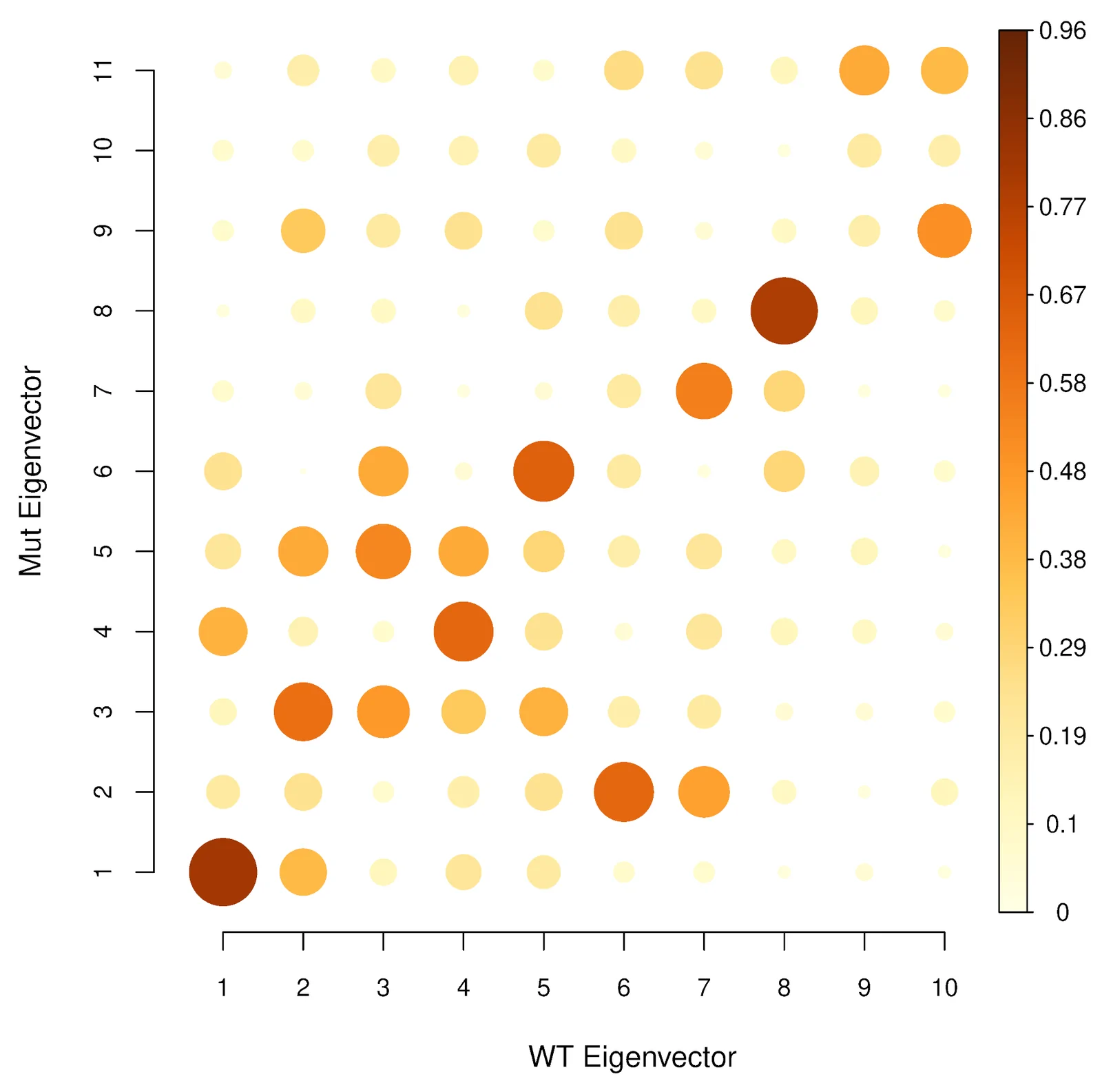
The root-mean-squared inner product matrix of the top 10 principal components. Darker and larger circles indicate greater overlap. Overlaps are not cumulative and do not sum to 1.0.

The normalized overlap of the covariance matrices, which ranges from 1 for identical matrices and 0 for orthogonal ones, is 0.625. Figure A8 shows the root-mean-square inner product matrix for the first 10 principal components of the wild-type and mutant eigenvalue matrices. The individual entries in this matrix reflect the degree of overlap between the given principal components. It can be seen that the first PC has a high degree of overlap, approximately 82%, meaning that, while the mutation does alter the greatest component of the collective dynamics, it leaves it mostly intact. On the other hand, while PC 2 of the wild type does have some overlap with PC 2 of the mutant, roughly 24%, it has the largest degree of overlap with PC 3 (60%) and a fair degree of overlap with mutant PC 5 (43%). Mutant PC 2 is most similar to wild-type PC 6, however, with an overlap of 62%. The trend continues with wild-type PC 3 having the greatest overlap with mutant PC 5 (53%), although it does have significant overlap with mutant PCs 3 (48%) and 6 (43%). Wild-type PC 4 is broadly similar to mutant PC 4 (62%), but wild-type PC 5 is more like mutant PC 6 (65%) than mutant PC 5. Together, Figure A7 and Figure A8 show that there is much similarity between the dynamic motions of the wild-type and mutant forms: wild-type components 1, 2, 4, and 5 have close or reasonably close mutant counterparts. However, the weighting of the first component is dramatically lower in the mutant form, describing almost 20% less of the total motion than the corresponding wild-type component. This has caused a rise in the contribution of the other components, making the S616L mutant more conformationally labile, as also reflected in the clustering results shown above.

**Figure A9:**
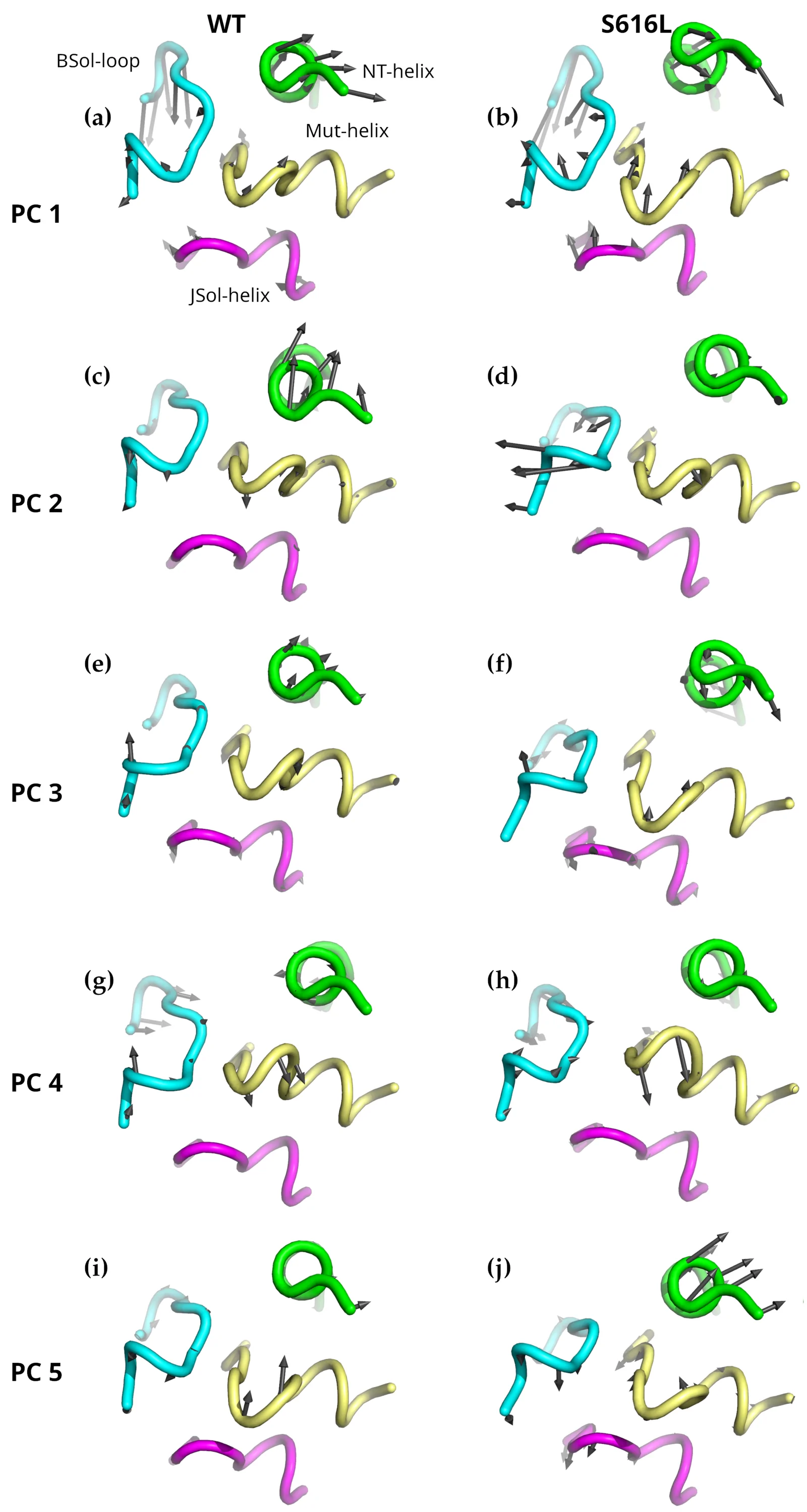
The Cα traces showing the extreme positions of the first five principal components. Each panel is colored as in Figure 1b. (**a**,**b**) show the first principal component for the wild type and mutant, respectively. (**c**,**d**), (**e**,**f**), (**g**,**h**), and (**i**,**j**) show, respectively, the wild-type and mutant traces for the second, third, fourth, and fifth PCs. The dark gray arrows indicate the directions of motion of individual Cα atoms that move more than 1.0 Å.

The dynamics of the first five PCs are illustrated in Figure A9, which shows the magnitude and direction of the characteristic motion captured by each PC starting from one of the extreme positions. The magnitude and direction of motion of the first PC are generally the same for both the wild type and the mutant (Figure A9a,b). The motion can be generally described as a clockwise rotation of the BSol-loop with respect to the other three interface loops; it can also be thought of as the vector that transforms the conformation described by cluster 1 above into the conformation described by cluster 2; it has about 83% overlap with the principal component vector that describes the difference between the wild-type and mutant trajectories illustrated in Figure A5b. The largest differences between the wild-type and mutant PCs are in the greater magnitude of motion in the Mut-helix in the mutant form and in a slight change of direction in the motion of the NT-helix.

In the second PC, the situation is quite different (Figure A9c,d). In the wild-type form, this component describes a motion in which the NT-helix and the other three loops move towards or away from each other, alternately opening and closing a gap between the NT-helix and the loop at the end of the Mut-helix (mut-loop). In the mutant form, the motion is considerably different. Here the N-terminal end of the BSol-loop and the other three loops move away from each other, with the BSol-loop making by far the larger movement. Comparing mutant PC2 to the clustering results suggests that it likely describes the formation of the conformation described by the S616L-exclusive cluster 4.

The third PCs are somewhat similar to one another, both describing the opening of a gap between the NT-helix and the Mut-helix. The main difference is in the direction of motion of the NT-helix. In the wild type, it moves directly away from the Mut-helix, while in the mutant form, it moves parallel to it. Both the wild-type and mutant PC3 feature a large motion of the N-terminal end of the BSol-loop parallel to the loop at the end of the Mut-helix (Figure A9e,f).

As expected from the inner product matrix, PC 4 of the wild type and S616L mutant are quite similar. The main difference is a slightly greater magnitude for the mutant PC. This PC describes the movement of the NT-helix and residues 620–623 of the Mut-helix away from each other together with the movement of the C-terminal end of the BSol-loop towards the Mut-helix. Comparing this motion to the clustering results suggests that this PC generally describes the transition between the conformation described by cluster 1 and that described by cluster 5 (Figure A9g,h).

The motion described by wild-type PC 5 is a small counter-clockwise rotation of the BSol-loop with respect to the Mut-helix and a larger motion by residues 620–623 of the Mut-helix. Mutant PC 5 is very different, being largely characterized by a sharp motion of the NT-helix away from the other three interface loops (Figure A9i,j).

Overall, PCA shows that the mutation largely preserves the main component of the dynamic motion while reducing its overall amplitude, thereby promoting the contributions of some of the smaller components, making the mutant form more conformationally labile.

#### 2.1.6. Effects on the Secondary Structure

**Figure A10:**
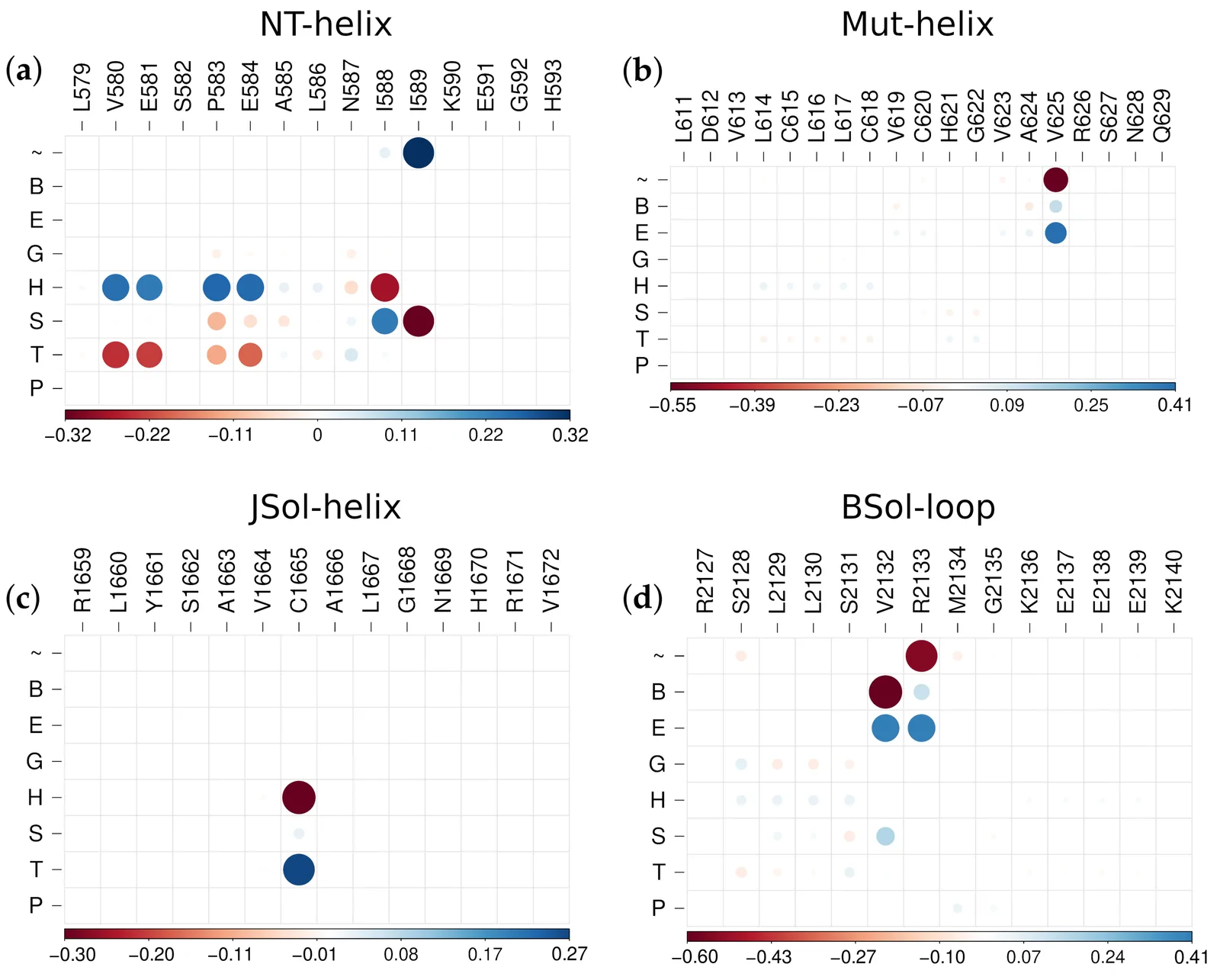
Matrices showing the changes in secondary structure as a result of the mutation in the interface loops, (**a**) NT-helix (**b**) Mut-helix (**c**) JSol-helix (**c**) BSol-loop. Red indicates a loss of the given secondary structure element for a particular residue as a result of the mutation, while blue indicates a gain. The larger and darker the circle, the greater the change. The secondary structure elements are denoted according to the DSSP standard and are designated as follows: ∼—loop, B—β-bridge, E —extended β-sheet, G—$3_{10}$ helix, H—α-helix, S—high curvature bends, T—hydrogen-bonding turns, and P—poly-proline II helices (the implementation of DSSP in Gromacs does not find π-helices by default, and very few were detected when checked).

Examination of Figure 2, Figure A2, and Figure A9 shows that the BSol-loop lies next to the mut-loop in a way that would make parallel β-strand hydrogen-bonding possible. It is therefore reasonable to ask what effect the mutation has on the hydrogen-bonding in this area. In particular, residues 618 and 625 and 620 and 623 might be expected to form main-chain–main-chain hydrogen-bonds as well as 626 and 2133, 624 and 2132, and 624 and 2131. Table A1 lists a number of relevant hydrogen-bonds, which shows that overall the mutant somewhat decreases the inter-strand hydrogen-bonds in the mut-loop and also decreases the main-chain–main-chain hydrogen-bonds between residue 2133 and residues 626 and 624. In compensation, the hydrogen-bond between residues 624 and 2133 has increased by more than 3×. On the other hand, using DSSP on the interface residues shows that the β-strand character of residues 619, 620, and 623 is slightly higher in the wild type, but the mutant has greatly increased the β-strand character of residues 625, 2132 and 2133 (Figure A10). The discrepency between these results arises from the fact that DSSP uses both backbone torsion angles and hydrogen-bonding patterns to assign secondary structure, thus some residues with appropriate ϕ,ψ angles may still be assigned as β-strand or β-bridging residues even if their total hydrogen-bonding contribution is lower. If we accept the verdict of DSSP, then it can be seen that the mutation increases the β-strand character of the Mut-loop–BSol-loop interface.

Figure A10 also shows that the mutation has effectively increased the α-helical character of the NT-helix by reducing its number of hydrogen-bonded turns and high curvature bends. In particular, residues 580 and 581 at the end of the preceding α-helix have increased their α-helical character by about 20%, while residues 583 and 584 at the beginning of the NT-helix have increased their α-helical character by 24%; residues 585 and 586 in the middle of the helix have increased their α-helical character by a more modest 5% or so, while residue 588 at the C-terminal end of NSol has become substantially less α-helical and residue 589 has shifted from a high-curvature bend to an unstructured loop. Overall, it appears that the part of NSol closer to the mutation site has become more ordered, while the part farther away has become less ordered. Interestingly enough, the mutation has also slightly increased the α-helical content of the residues surrounding the mutation site.

#### 2.1.7. Hydrogen-Bonding Analysis

These changes in the conformation of the interface loops also result in changes to the hydrogen-bonding patterns observed in the mutant. The largest changes in the H-bonds shared between both the wild-type and mutant forms are given in Table A2. Perhaps surprisingly, the mutant forms more hydrogen-bonds than the wild type (18.2 per frame versus 17.8) but has a smaller number of possible hydrogen-bonds (161 versus 167 for the wild type) even though the mutant lacks the Ser-616 Oγ atom. The increase in total hydrogen-bond number is mainly due to the increase in occupancy of five hydrogen-bonds between the BSol-loop and the Mut-helix, which is only partially balanced by a decrease in occupancy of two other bonds between the same groups. One of these is a β-bridge between Arg-626 and Arg-2133, which is countered by a decrease in a similar β-bridge between Ala-624 and Ser-2131 (the other two are side-chain–side-chain H-bonds between Arg-626 and Glu-2138). There has been an extensive remodeling of the hydrogen-bonds within the NT-helix, which has led to a net increase of about 2% in the total number of hydrogen-bonds within the NT-helix in the mutant. Examining the main-chain–main-chain hydrogen-bonds in Table A2 shows, consistent with the previous section, that the α-helical H-bonds near the N-terminal end of the NT-helix, 583–587 and 582–586, have increased in occupancy, while those near the C-terminal end, 585–589 and 584–588, have decreased. The occupancy of all $3_{10}$-helix H-bonds in the NT-helix (587–584, 582–585, 583–586, and 585–588) has decreased. Most of the H-bonds involving NT-helix side-chain atoms are to backbone atoms of the same helix, with only one side-chain–side-chain H-bond (between residues Ser-582 and Glu-584), and the mutant has increased the occupancy of these by a total of around 45%. The mutant form has also increased the occupancy of all the α-helical H-bonds on either side of the mutation site; that is, the H-bonds between residues 614–618, 615–619, and 613–617 have all increased (boldface entries in Table A2).

The H-bonds that exist in only one form but not the other are all very minor, with the most populated H-bond (a side-chain–main-chain H-bond from Nδ2 of Asn-587 to the backbone O atom of Arg-2133) being occupied in only 3.2% of the trajectory frames (Table A3). Many of the most highly occupied H-bonds in this table are side-chain–side-chain H-bonds between the NT-helix or the Mut-helix and the BSol-loop that are lost in the mutant form.

Overall the effect of the mutation has been to increase the number of H-bonds around the mutation site and between the BSol-loop and the Mut-helix while decreasing the number of H-bonds between the BSol-loop and the NT-helix.

#### 2.1.8. Effect of the Mutation on the NSol Domain

The mutation site occurs in the NSol domain, yet the largest conformational changes seem to be those of the BSol-loop as seen in the clustering analysis (Figure 2 and Figure A2), the RMSF analysis (Figure 3), the COM analysis (Figure A3), and the principal components analysis (Figure A9). The mechanism by which the mutation triggers these changes must therefore involve changes to the NSol domain that then lead to disruption of the NSol–BSol domain interface. Although not explored extensively here, we may note that the distances betweens the COMs of the whole BSol1 and JSol domains are notably smaller in the mutant structure and that the average amount of solvent accessible surface area buried by the JSol–BSol interface has increased in the mutant from 16.88 nm^2^ to 21.07 nm^2^. These increases have come at the expense of the NSol–BSol1 interface, which has an increase in whole domain COM distance and an overall decrease in buried solvent accessible surface area (from 6.71 to 6.11 nm^2^) in the mutant. The most reasonable conclusion is that the mutation somehow changes part of the NSol closest to to the BSol1–NSol interface, which thereby destabilizes it, leading the BSol1 domain to adopt a tighter interface with JSol.

Two of the changes to the NSol domain have been noted above: in the preferred mutant conformation, the relative RMSFs of the residues of the NT-helix and Mut-helix have decreased (Figure 3), while the occupancy of all the α-helical H-bonds on either side of the mutation site has slightly increased (boldface entries in Table A2) and the α-helical character of the NT-helix has been stabilized (Figure A10a).

**Figure A11:**
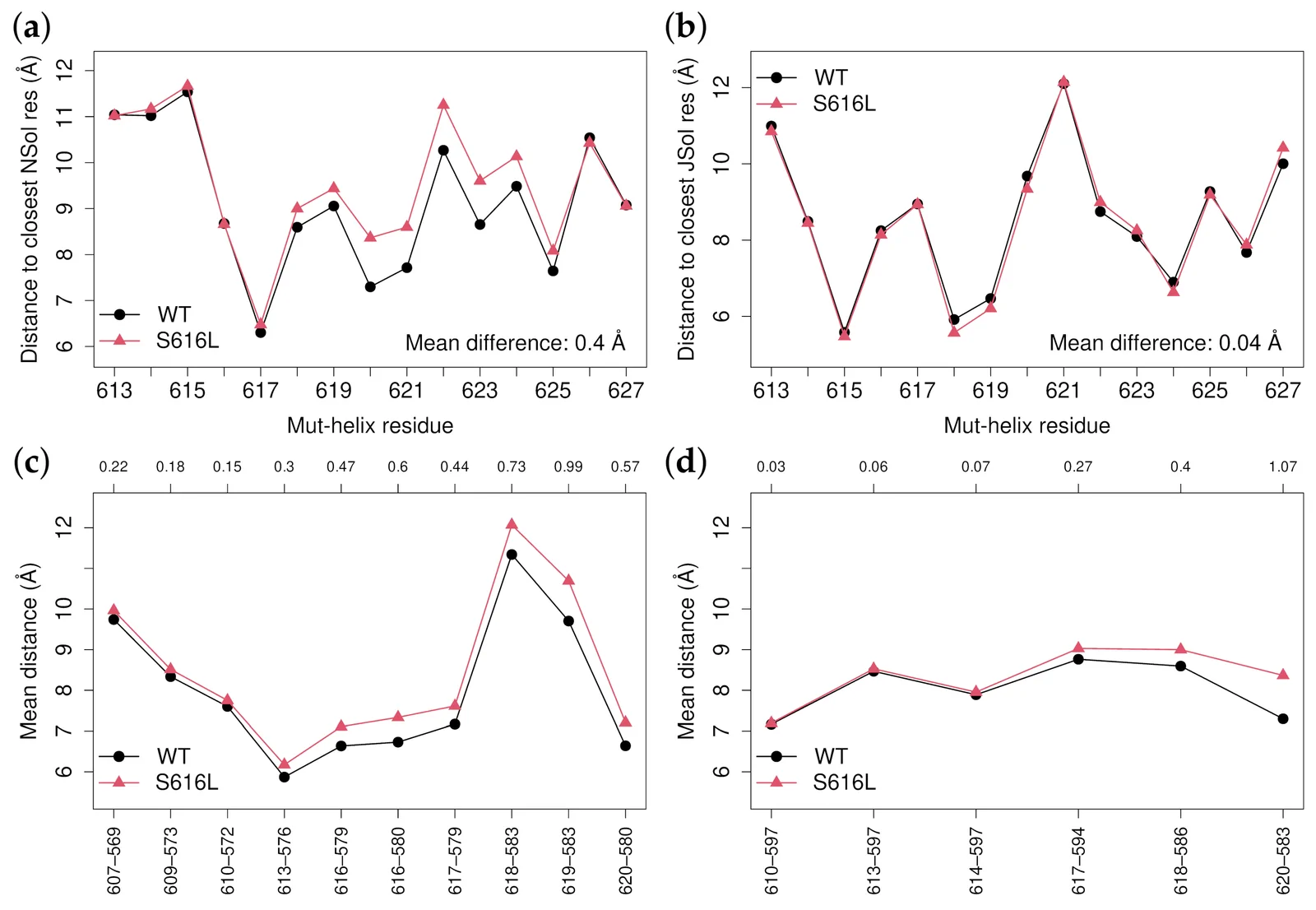
Plots showing Cα–Cα distances between residues in Mut-helix and the closest residues in the NT-helix (**a**) and the JSol-helix (**b**). The lower two panels show the mean Cα–Cα distances between the given pairs of residues where one residue is from the Mut-helix and the other is from the α-helix lying immediately above it ((**c**); the orange helix in Figure A12) and adjacent to one side ((**d**); the cyan helix in Figure A12). The black points show the wild-type distances, and the red triangles show the mutant distances. It can be seen that residues following the mutation site tend to be farther apart in the mutant.

**Figure A12:**
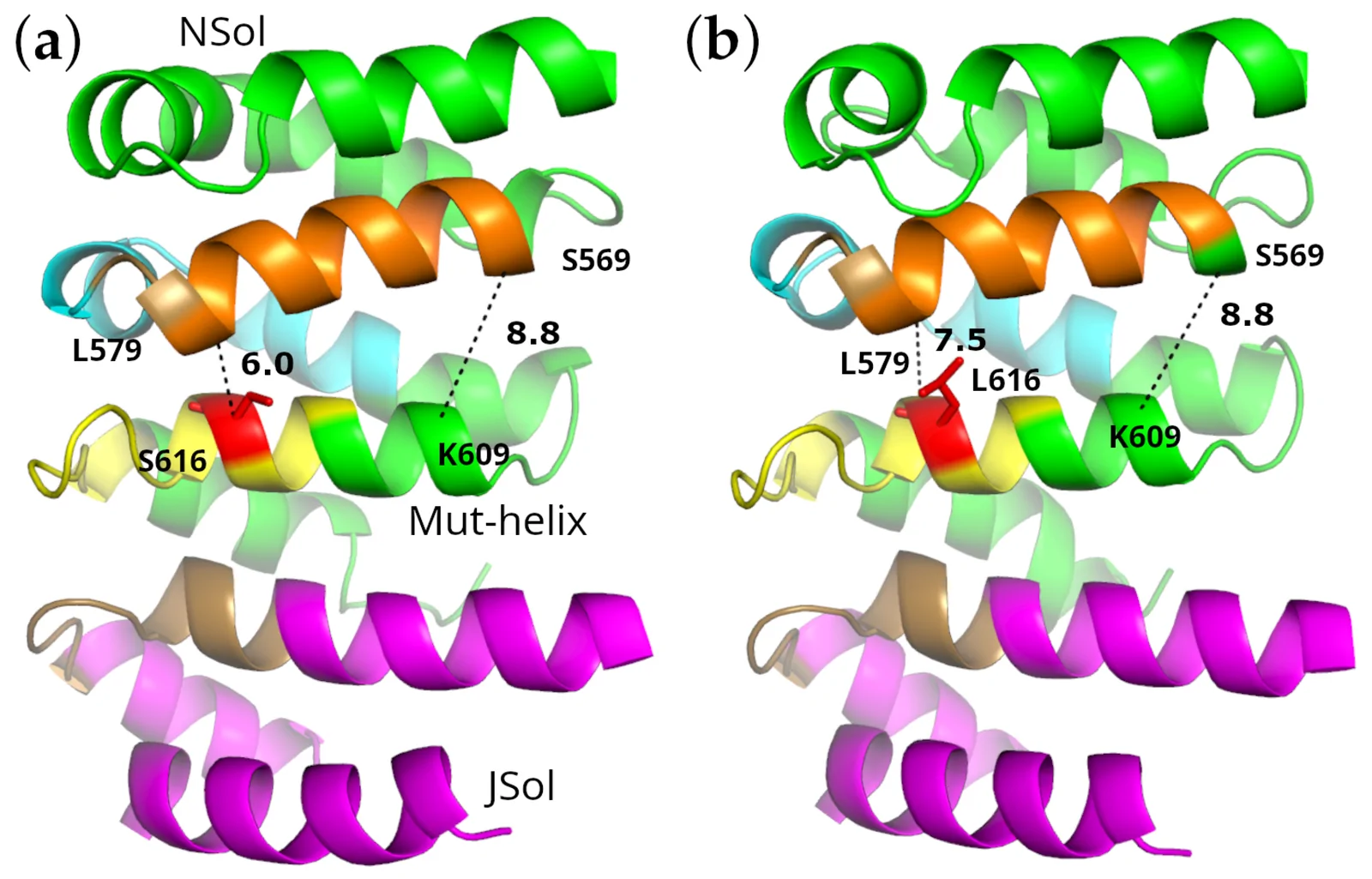
Fragments of the structures from the wild-type and mutant trajectories corresponding to the representative structures from (**a**) cluster 2 (wild type) and (**b**) cluster 1 (the S616L mutant). The fragments shown cover the residues within 25 Å of residue 616. It can be seen that there is a clear tilting away from the mutation site. Distances between selected pairs of Cα atoms from the Mut-helix (yellow) and the orange helix (residues 569–583) are given in Figure A11c, while those between selected pairs from the Mut-helix and the cyan helix are given in Figure A11d.

Another change can be seen in Figure A11a,b, which shows that the distances between the Mut-helix and the NT-helix and the Mut-helix and the JSol-helix actually increase in the mutant structure. Since leucine is a physically larger residue than the serine it replaces in the mutant, this is not unexpected. Figure A2 and Figure A12 compare the residues within 25 Å of residue 616 of two representative structures from the wild type and mutant. These structures correspond to the trajectory frames that formed the center of the dominant clusters of the wild type and S616L mutant from the clustering analysis above. It can be seen in this figure that the Mut-helix and the JSol-helix are almost identical between the two structures but that the residues on the NSol side of the Mut-helix have all shifted away from the mutation site. The structure also shows that the leucine substitution has caused a displacement of the entire helix–loop–helix motif containing residues 576–589. This can be seen more quantitatively in Figure A11c,d, which shows the Cα–Cα distance between individual residue pairs between the α-helix containing the mutation site and the two NSol α-helices closest to it. These pairs were selected by manually identifying the residues with the closest Cα–Cα distances from the wild-type structure shown in Figure A12. It can be seen that the distance to the first α-helix (orange in Figure A12) is relatively unchanged up to residue 610, but beginning at residue 612 (the residue that makes an n+4 α-helical hydrogen-bond with residue 616), the distance increases, reaching a maximum of nearly 1 Å at residue 619. Likewise, it can be seen that the distance to the second α-helix (cyan in Figure A12) is largely unchanged until after residue 616, when it also increases, again reaching a maximum of more than 1 Å at residue 620.

**Figure A13:**
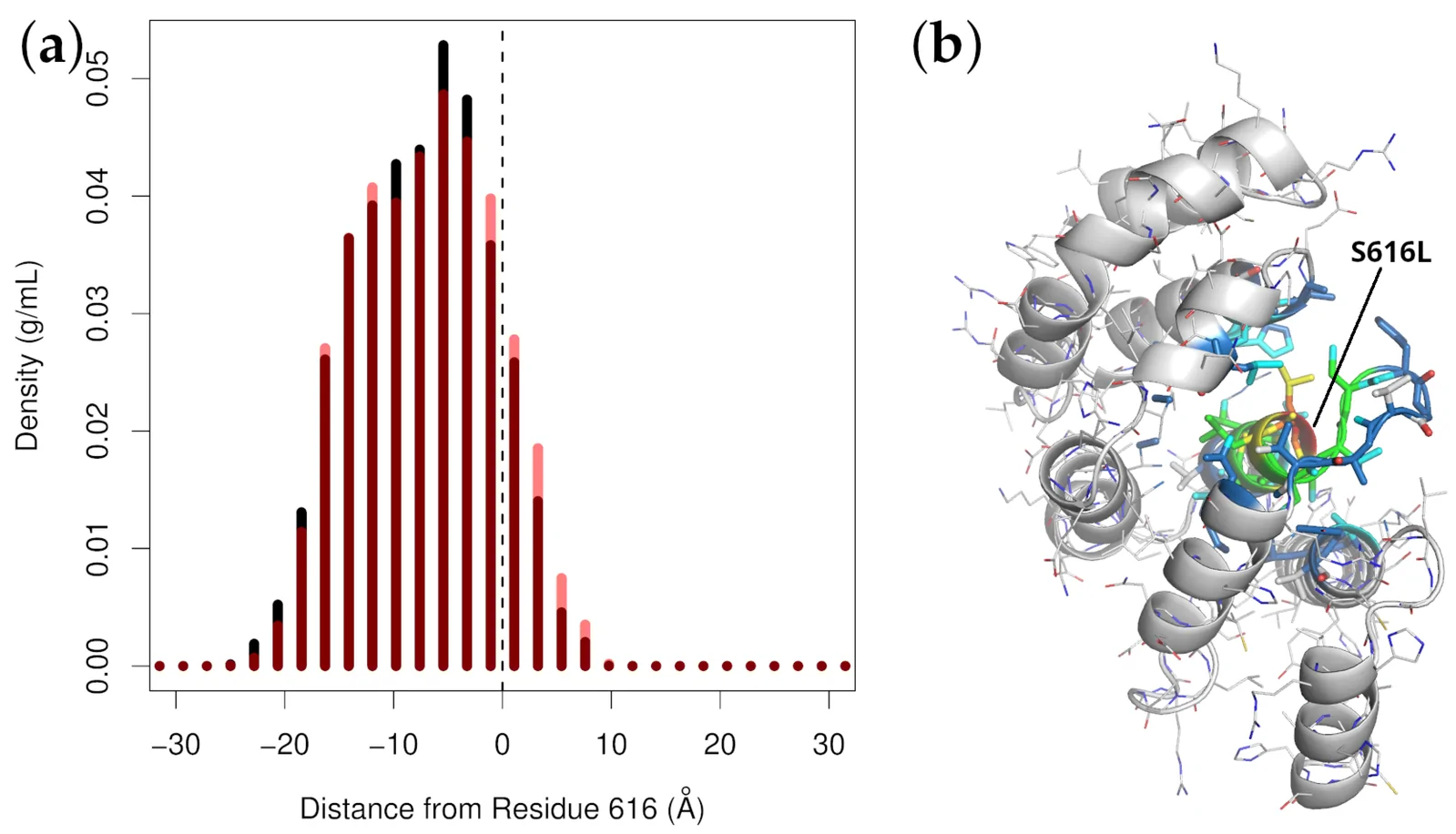
The change in NSol density caused by the mutation. (**a**) The density profile of the NSol domain from the wild-type (black) and mutant (red) fragments centered on the mutation site (dashed line at 0). The profile is along the *z*-axis, which corresponds to the view of the fragment shown in (**b**). Panel (**b**) shows the wild-type structure oriented so the *z*-axis is in the horizontal direction so that the fragment is in the same orientation as the density profile in (**a**). The atoms within 10 Å of the Cα atom of residue 616 are colored by distance (0 Å—red, 2 Å—orange, 4 Å—yellow, 6 Å—green, 8 Å—cyan, 10 Å—blue, greater than 10 Å—white).

This increase in distance has had the effect of reducing the density of the NSol domain within approximately 10 Å of the Cα atom of residue 616 (Figure A13) but increasing the density beyond that. The overall effect is to slightly increase the density of the mutant NSol domain with respect to the wild-type NSol domain, but there is still a lower-density area immediately around the mutation site.

This shift in packing may be the cause of the increase in overall RMSF observed in the NSol domain. The greatest increase has occurred in a part of the domain far from the interface, but the area within 25 Å of the mutation site has also shown a notable increase in RMSF. This change in density may also be responsible for the change in the preferred dynamic motion of the NT-helix described above. Thus, the effect of the S616L mutation on the NSol domain appears to be to force a change in the conformation, flexibility, and dynamics of the residues immediately surrounding the mutation site, most especially the residues comprising the NT-helix. This, in turn, disrupts the NSol–BSol interface, causing BSol to interact more tightly with the JSol domain.

#### 2.1.9. Molecular Dynamics Summary

In summary, the S616L mutation introduces a subtle change to the overall conformation of the NSol side of the three-domain junction. This change, though relatively small, appears to cause the mutant form to adopt a different preferred conformation, as seen in the RMSD and clustering analyses. The new preferred conformation rotates the BSol-loop approximately 18° in a clockwise direction with respect to the other three interface loops, thereby moving the BSol1 domain away from the NSol domain towards the JSol domain. The new conformation has a slightly lower RMSF in nearly all residues on the NSol side of the interface, a slightly higher one on the JSol-helix side, and a split in the BSol-loop. PCA together with the clustering and RMSD analyses also show that the S616L mutation has modestly increased the conformational variability of the interface, mostly in the BSol-loop and the NT-helix. These shifts also resulted in changes to the secondary-structure content of the interface, its hydrogen-bonding properties, and its preferred dynamic motion. All these changes suggest that the mutation has effectively weakened the interface between the NSol and BSol domains, which would promote the formation of the opened form of the receptor.

### 2.2. Expression and Purification

**Figure A14:**
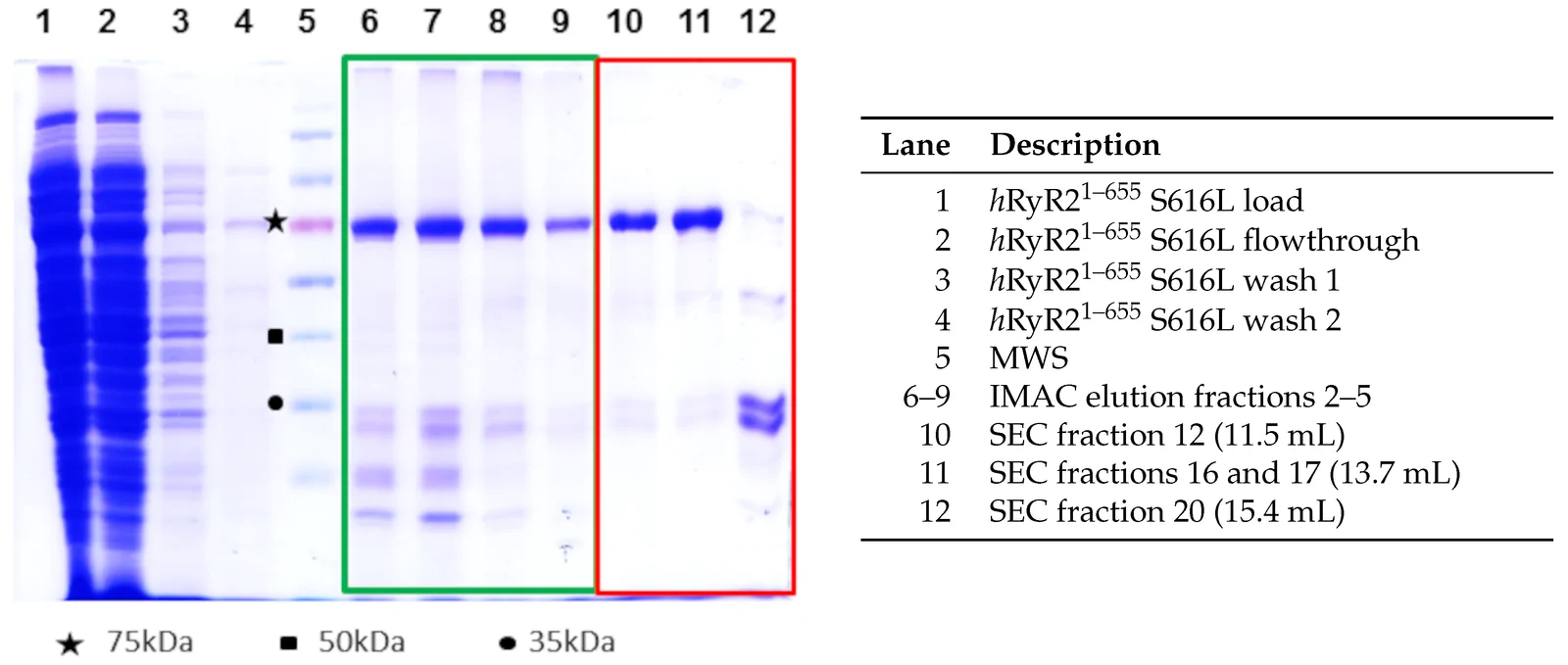
Purification of the *h*RyR2^1–655^ NTD S616L mutant by IMAC and SEC. 15% SDS-PAGE. Lanes: 1. Soluble fraction after sonication and centrifugation of BI21 (DE3) with expressed *h*RyR2^1–655^ NTD S616L mutant; this was loaded on the IMAC column; 2. Flowthrough; 3–4: Wash; 5. MWS (Fisher Scientific, 26 616); 6–9. Elutions E2 to E5 after IMAC purification (green box); 10–12. Fractions after SEC purification (red box). Lanes 10 and 11 show fractions 12 and 17 from SEC, and they represent the oligomeric and monomeric forms of the *h*RyR2^1–655^ S616L protein, respectively. All volumes mark the center of the collected fraction.

**Figure A15:**
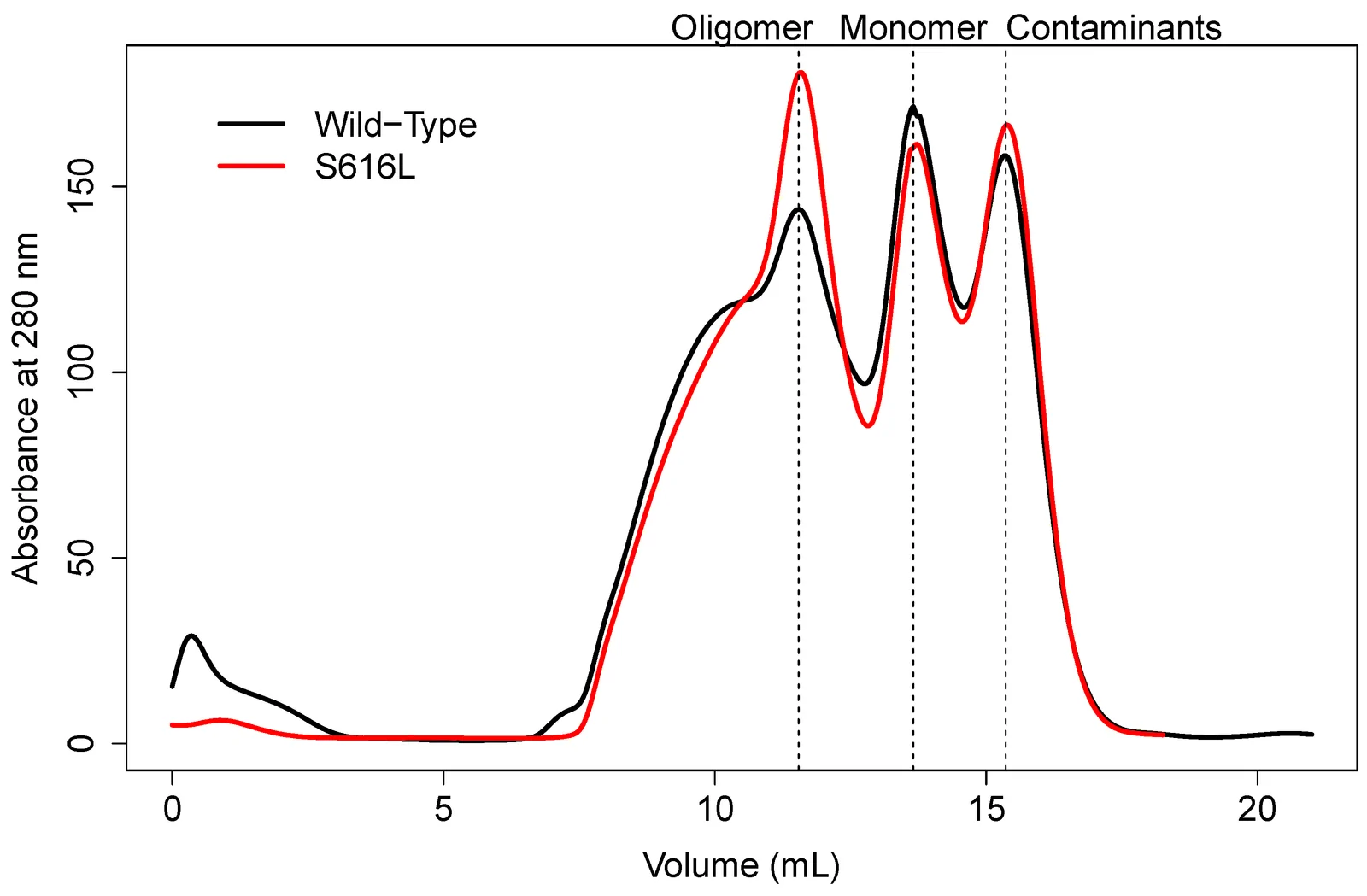
SEC Chromatogram of wild-type *h*RyR2^1–655^ NTD and the S616L mutant. The monomeric form of both proteins (MW ≈ 74 kDa) eluted at ≈13.7 mL. Peaks 1 to 3 correspond to the SDS-PAGE gel Figure A14, lanes 10–12. BSA (MW ≈ 68 kDa) was eluted at 14.0 mL.

**Figure A16:**
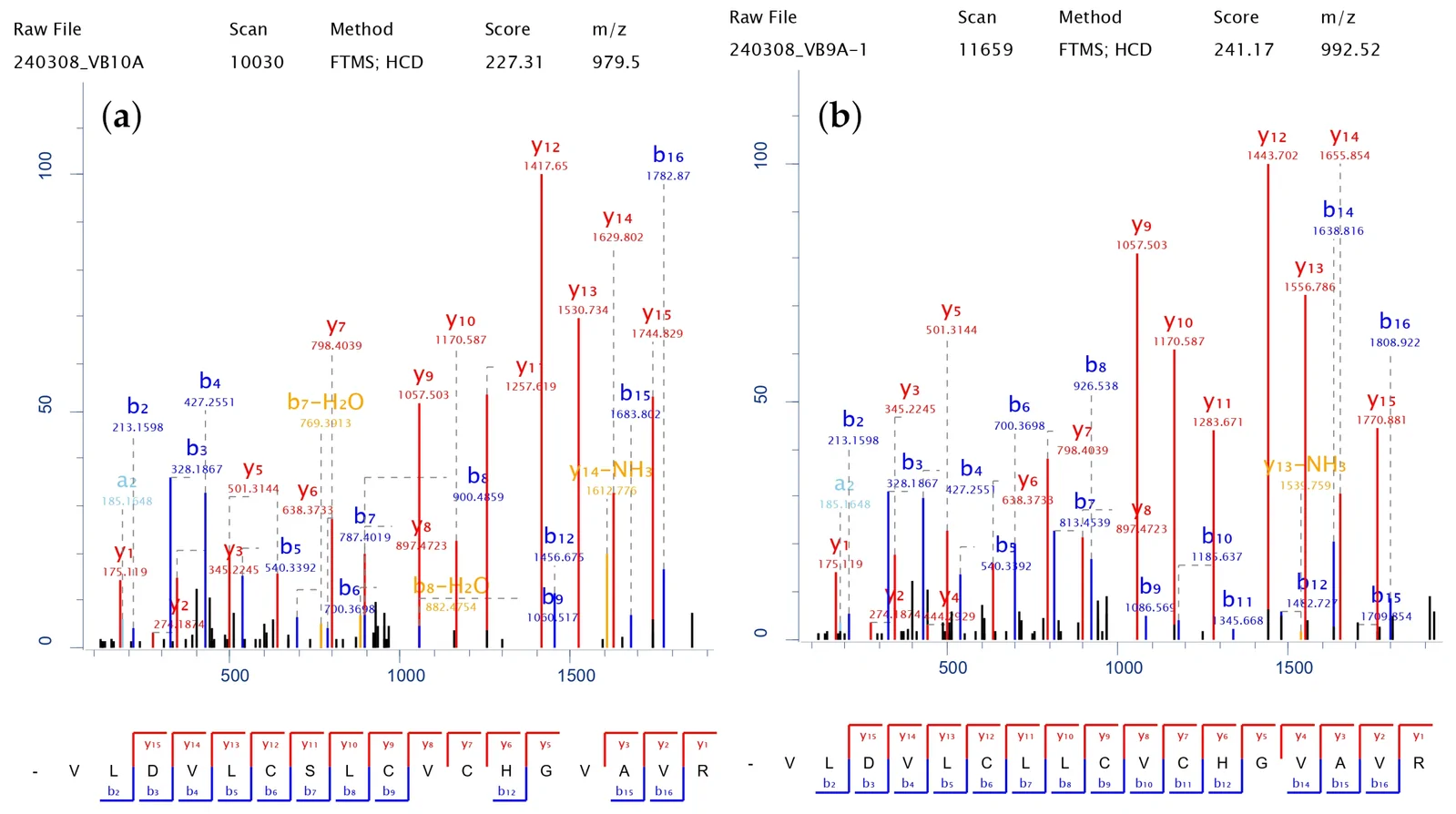
MS/MS spectra of the wild-type (**a**) and mutant (**b**) fragments of *h*RyR2^1–655^, confirming the presence of the S616L substitution.

To study the effect of the S616L mutation biophysically, we cloned, expressed, and purified a fragment of human RyR2 containing the first 655 residues (*h*RyR2^1–655^). Expression levels of wild-type *h*RyR2^1–655^ and the S616L mutant were similar, ranging from 0.5 to 0.8 mg of purified protein per L of culture media. Both proteins could be purified to >95% purity (Figure A14) using IMAC and SEC. Both proteins could be isolated in two forms, a monomeric form and a higher oligomeric form (size according to SEC). The monomeric forms of both *h*RyR2^1–655^ and the S616L mutant were separated by FPLC gel filtration (Figure A15) calibrated with BSA, Conalbumin, and aldolase. The monomeric form of the pure *h*RyR2^1–655^ NTD fragment eluted at ≈13.7 mL, which is similar to BSA and Conalbumin, which eluted at 14.0 mL and 14.4 mL, respectively. The larger elution volumes of the wild-type and mutant *h*RyR2^1–655^ NTD fragments were likely due to the known tendency of the C-terminal end of the NTD to become unwound [49]. Only the monomeric form of both proteins was further characterized. The identity of the fragments was confirmed using LC–MS/MS (Figure A16), which also showed that both proteins were very pure. Both proteins were characterized using circular dichroism (CD) spectroscopy and nano-differential scanning fluorimetry (nanoDSF).

### 2.3. CD-Spectroscopy

**Figure 4:**
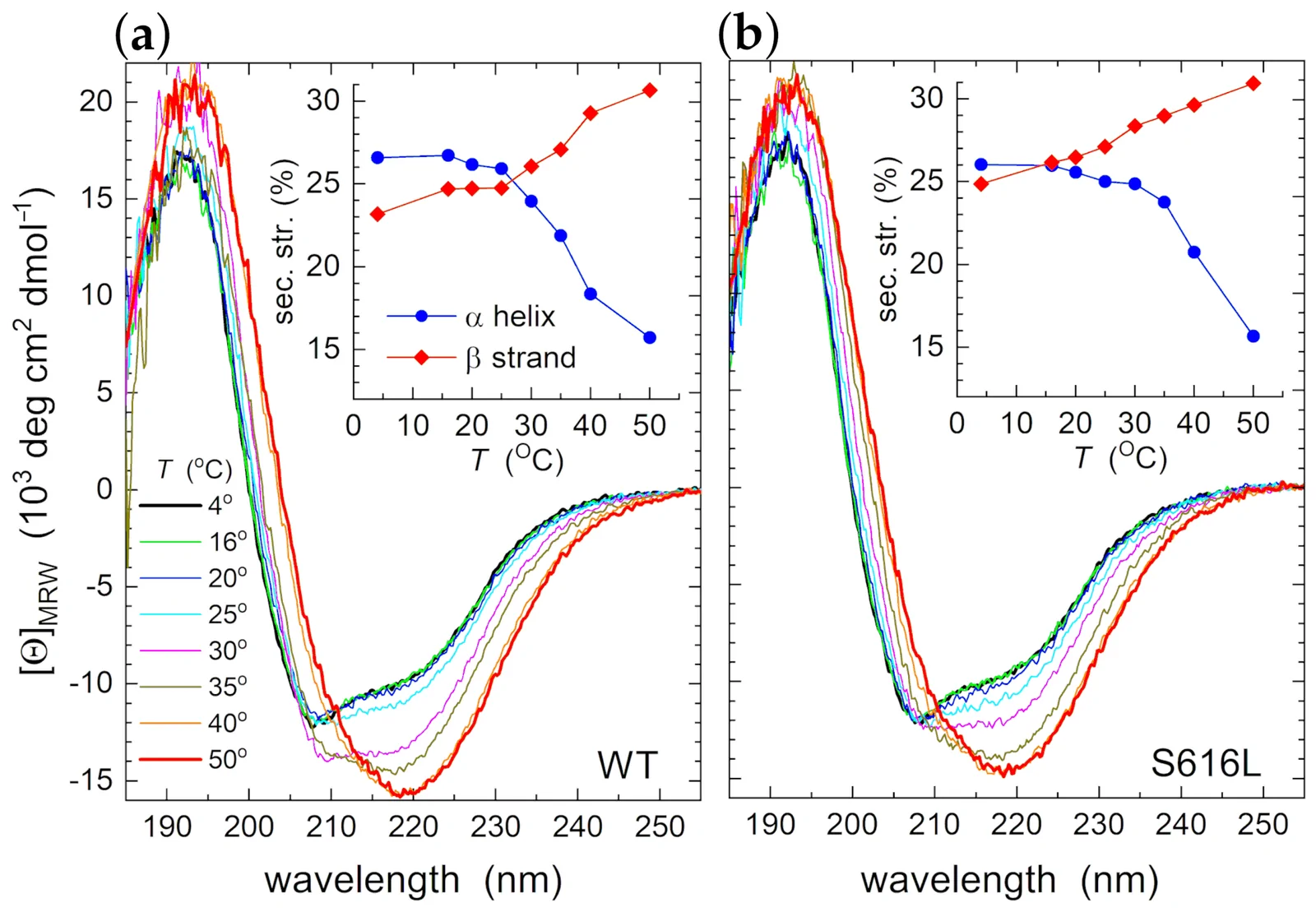
CD spectroscopy of (**a**) monomeric wt-*h*RyR2^1–655^ and (**b**) its S616L mutant measured at 4 °C (black), 16 °C (green), 20 °C (blue), 25 °C (light blue), 30 °C (magenta), 35 °C (olive green), 40 °C (orange), and 50 °C (red). The insets show changes in the secondary structure element content (α-helices (blue), β-sheets (red) with respect to the temperature increase.) The spectra shown are from a one-time measurement that is representative of a series of three.

The CD spectra of wt *h*RyR2^1–655^ and the S616L mutant were recorded at the following temperatures: 4, 16, 20, 25, 30, 35, 40, and 50 °C (Figure 4). The spectra exhibit the characteristics of a folded protein containing α-helices and β-strands. Surprisingly, the wild-type fragment was less stable than the mutant, with large differences between 25 °C and 30 °C at 208 and 220 nm. At temperatures above 30 °C, the pronounced minimum at 208 nm and shoulder around 220 nm that indicate α-helices were gradually shifted to about 218 nm, and the overall spectra showed a bathochromic shift suggesting an increase in β-strands. Such a change is supported by a deconvolution of the spectra, which showed that the α-helical and β-strand content was 26–27% and 22–25%, respectively, at 4 °C, but at 50 °C, the corresponding amounts were 12–19% and 27–31% range (Figure 4, insets). Visual inspection showed that the decrease in α-helicity observed for all samples in the 25–35 °C range is accompanied by the development of turbidity. No renaturation could be observed upon cooling from 50–4 °C.

### 2.4. nanoDSF

**Figure 5:**
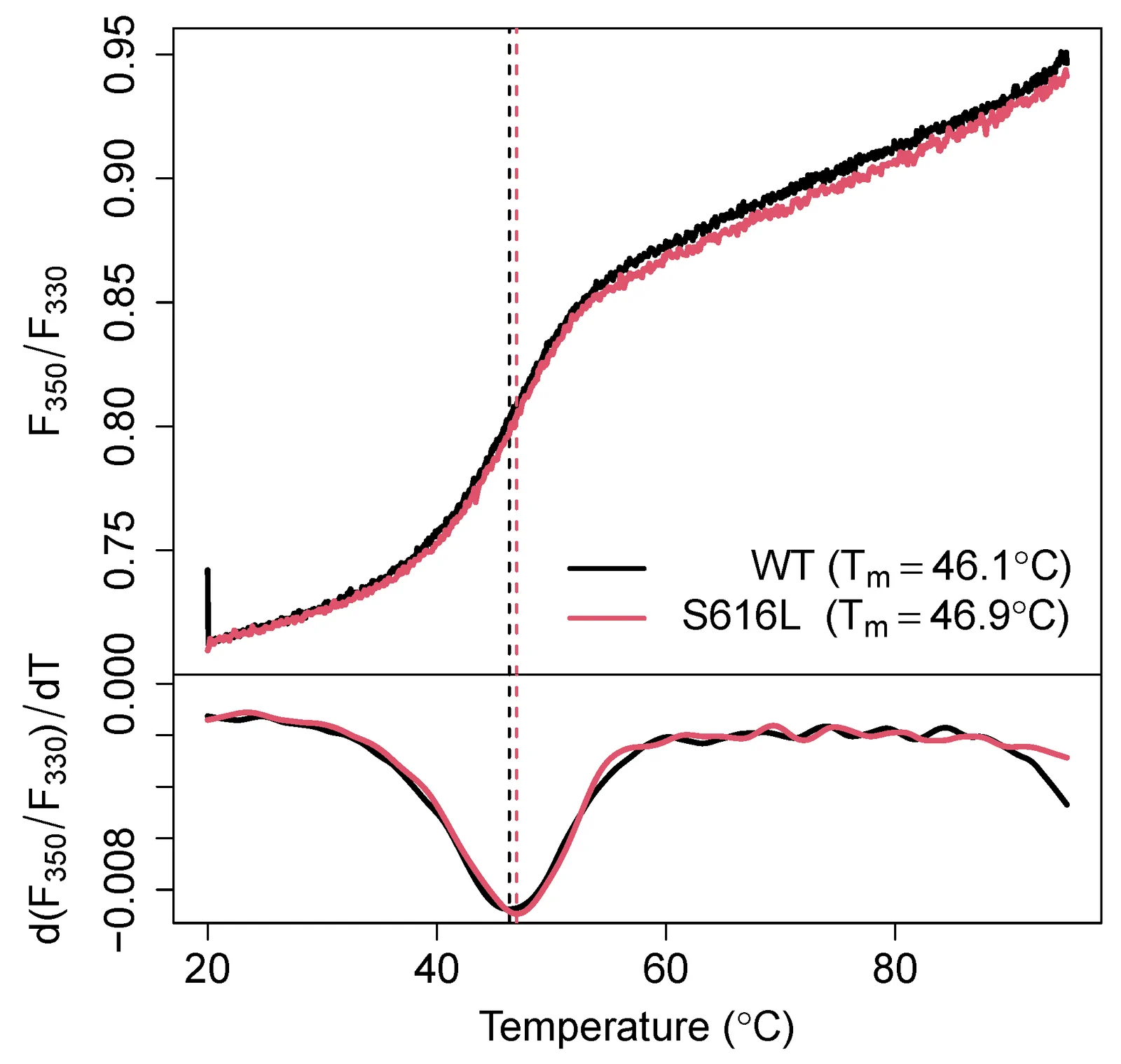
NanoDSF thermal unfolding curves of wt *h*RyR2^1–655^ and the S616L mutant. The ratio of the fluorescence observed at 350 nm and 330 nm is shown at top ($F_{350}/F_{330}$), and its first derivative is shown at bottom ($d(F_{350}/F_{330})/dT$). The vertical dashed lines mark the inflection points of the corresponding unfolding curves, indicating the T_m_ of each protein. Only the monomeric form of each protein was measured.

To more precisely determine the denaturation temperature of each fragment, we employed nanoDSF [50]. The denaturation curves of the monomeric form of each protein were measured over a range from 20 to 85 °C, and the melting temperature T_m_, where 50% of the molecules are denatured, was determined from the inflection point of the curve. This is most conveniently determined by identifying the highest peak in the first derivative of the curve. NanoDSF showed that denaturation began between 33 °C and 35 °C for both fragments. The S616L mutation very slightly increased thermal stability of the fragment: wild-type *h*RyR2^1–655^ had a T_m_ of 46.1 ± 0.4 °C, while the S616L mutant was higher at 46.9 ± 0.2 °C (Figure 5A).

## 3. Discussion

It has been proposed that Ca^2+^ leaks from defective RyR channels as a consequence of domain “unzipping,” that is, in the closed form, two or more domains make close contacts that stabilize the closed or “zipped” form, while pathogenic mutations can weaken these interactions, causing the interdomain interface to loosen [38,39]. A number of recent cryo-EM structures of both *h*RyR1 [13,40] and *h*RyR2 [12] have confirmed this hypothesis in a general way, showing, for example, that R2474S in *h*RyR2 and Y523S in *h*RyR1 both subtly alter the interfaces between the NTD and its flanking domains to produce structures that, in the closed state, are already partially in the open state, thereby lowering the activation energy for opening and increasing the opening probability.

In this context, residues 545–640 of the *h*RyR2 NTD form an attractive target for study (Figure 1). This region forms the meeting point for the SPRY domains on one side and the N-terminal end of the BSol domain on the other. From the N-terminal edge, it borders the rest of the NTD, while from the C-terminal edge, it forms an extensive interaction with the JSol domain. It even forms a minor part of the FKBP12.6 (calstabin-2) binding site in all the cryo-EM structures determined to date. In particular, a loop comprising residues 86–93 of calstabin-2 lies in a shallow groove formed by the last NTD α-helix (*h*RyR2 residues 634–639) and the second JSol α-helix (residues 1671–1679). Arg-640 also forms a weak H-bond to Phe-37 of calstabin-2. Until recently, this region had also been thought to be the binding site for the muscle relaxant dantrolene [51,52], perhaps sensibly given its central location but questionable given the lack of any obvious binding pocket formed by the subdomain itself. Interestingly, despite its appearance as a well-folded armadillo-repeat domain, SAXS studies of this region show that it is largely unfolded when expressed as part of the NTD alone, and MD simulations show that its motion is largely uncoupled from that of the rest of the NTD [49,53]. All this suggests that this region is at least somewhat conformationally labile but is held in place by interactions with the neighboring domains.

**Figure A17:**
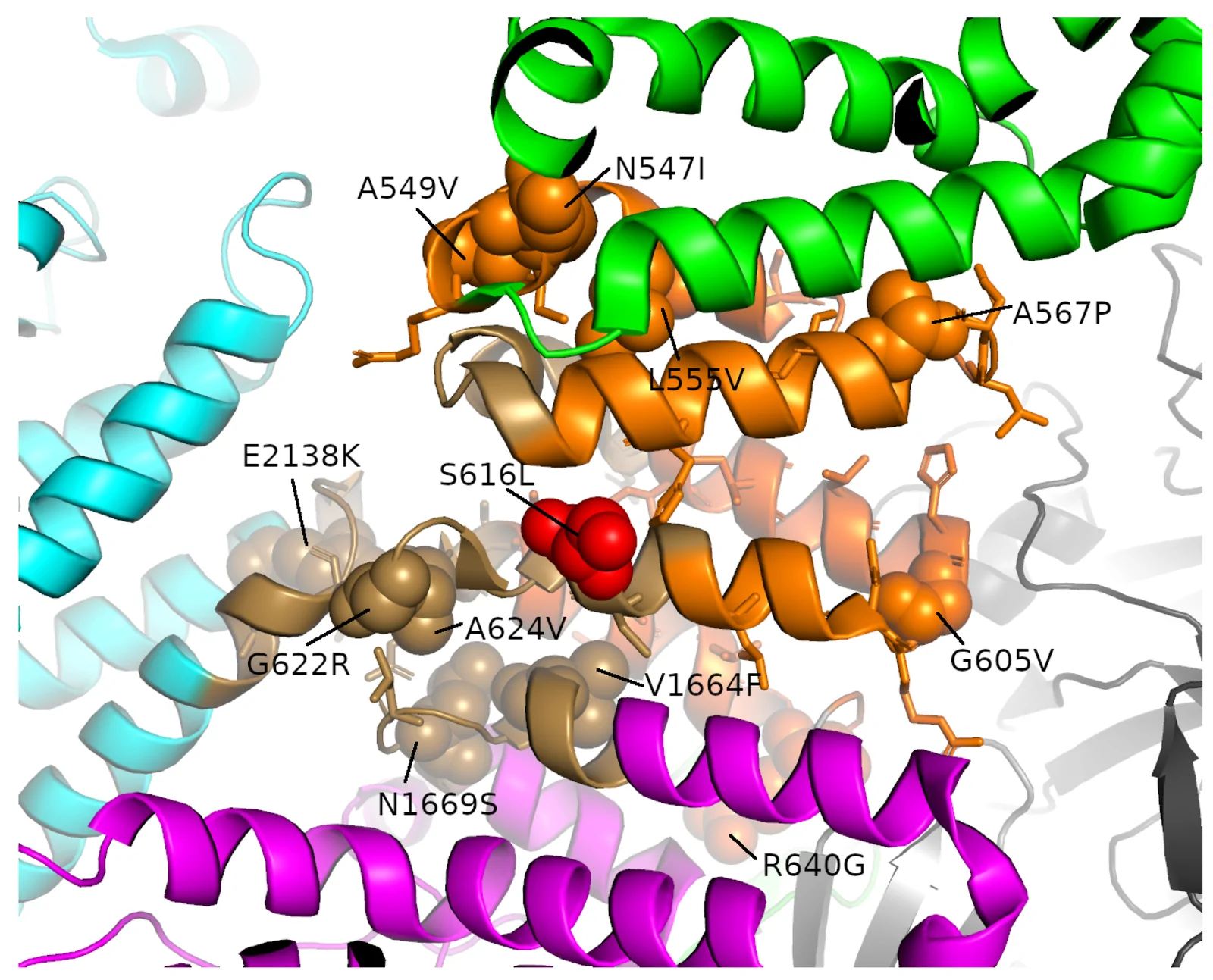
View of the region surrounding the S616L mutation colored as in Figure 1. All residues whose mutations have been associated with cardiac myopathies (by clinical observation or publication according to ClinVar [54]) are shown as spheres; those suspected but not shown to cause myopathies are shown as sticks.

ClinVar [54] holds records on 80 clincally observed variants in 60 different sites in this region. Of these, eight have been established to be pathogenic, while another 23 are predicted to disrupt the protein with varying degrees of certainty (Figure A17, Table A4). Of the pathogenic ones, all but two of them are located at the interface between this region and one of the neighboring ones: N547I [55], A549V [22,56,57], and A567P [58,59] are located at the interface between this region and the rest of the NTD solenoid; G605V (unpublished, ClinVar Accession 2737123) contacts SPRY3; G622R [60,61] and A624V [62] form part of the Bsol1 interface and also interact with residue 1667 from JSol (itself the site of a possibly pathogenic mutation); and R640G [62] both interacts with SPRY1 and forms part of the FKBP12.6 binding site. The two exceptions that are not directly part of an interface are L555V [63] near the center of the domain and S616L [33], which is close to the JSol and BSol1 interfaces but is not itself part of them.

Since S616L lies exactly at the area where three domains come together, we thought it worth exploring the effect of this mutation on the structure and dynamics of this region. To this end, we carried out a limited exploration on only those areas in the immediate vicinity of the S616L mutation site. The main effect of the S616L mutation is to alter the preferred conformation of the BSol1–NSol interface. It appears to do this by increasing the ordering of the α-helix containing the mutation site, decreasing the density of the NSol domain in the immediate neighborhood of the mutation site, and inducing a subtle conformational change that disfavors the interactions between the NT-helix and BSol-loop seen in the wild-type form in favor of a closer interaction between the BSol1 and JSol domains. This conformational change is accompanied by a decrease in the RMSF of most of the residues on the NSol side of the interface but an increase in the RMSF of the NSol domain more generally. Principal components analysis suggests that the mutation decreases the amount of dynamic variability captured by the first principal component, allowing more for the other components and thereby complicating the dynamic landscape. The root-mean-square inner product matrix of the covariance matrices together with an examination of the first five principal components shows that the mutation has partially inhibited the largest motions in the wild type while increasing the contributions of some smaller motions. The mutation has also resulted in an increase in the number of hydrogen-bonds in the interface, mostly by increasing the occupancy of five of them between the BSol-loop and the Mut-helix. Overall, the simulation suggests that the S616L mutation shifts the position of the NT-helix, thereby promoting the shift of the BSol-loop. NanoDSF and CD-spectroscopy also showed that the S616L mutation slightly increased the thermal stability of the *h*RyR2^1–655^ fragment, which is consistent with the mutation increasing the ordering of the area around the mutation site. By weakening the NSol–BSol1 interface, the mutation could plausibly lead to the type of primed, partially open conformation similar to those previously observed for the R2474S mutant in *h*RyR2 [12] and Y523S mutant in *h*RyR1 [40].

The importance of the NTD–BSol1 interface is further highlighted by the fact that at least three pathogenic mutations have been documented to occur at it: G622R [60,61] and A624V [62] from the NTD, E2138K from BSol1 (unpublished, ClinVar accession 532366), and N1669S [64] and V1664F [65,66] from the JSol domain. In addition, R626C (ClinVar accession 3623525), L1667P (924339), and V2132M (3385762) are predicted to be disruptive, though they have not been clinically shown to be pathogenic. Superimposing the open and closed conformations by explicitly aligning residues 545–640 shows that there are very few differences in the immediate area of the interface, but there are larger ones a short distance away. Viewed from the inside surface, the NTD moves almost not at all, while the JSol and BSol1 domains undergo a small counterclockwise rotation, which ultimately results in the downward and outward shift of the cytoplasmic shell described earlier [12]. The weakening of this interface might be expected to cause BSol1 to shift at least partially towards its open conformation, thereby putting the protein into a “primed” state, similar to those observed for other mutants.

## 4. Materials and Methods

### 4.1. Molecular Dynamics Simulation

It was not feasible to carry out MD simulation on the entire *h*RyR2 using the computational resources at our disposal. We therefore restricted ourselves to one particular question: what is the effect of the S616L mutation on the structure and dynamics of the residues in the interdomain contact area surrounding the mutation site? Answering this question should highlight the importance of this site and what sorts of interactions are important during the closed state. To address this question, we created a structure from chain A of PDB structure 7ua5 [12] (representing the closed form of *h*RyR2 without any additional modifiers) containing residues 409–647, 1641–1763, 1777–1854, 1889–2010, 2053–2364, 2377–2444, and 2455–2476. These residues create a region containing all of the α-helical solenoid of the NTD (NSol), nearly all of the JSol domain, and almost 2/3 of the BSol1 domain. The S616L mutant was prepared from this fragment by introducing the corresponding point mutation in PyMOL (version 3.1.0) [67].

MD simulations were carried out using GROMACS 2023.2 [68] with the CHARMM36m force field [69]. Each structure was placed in a rhombic dodecahedral box with a 1.2 nm cushion, which was then filled with TIP3P water [70]. Periodic boundary conditions were used, and an appropriate number of Na^+^ and Cl^−^ ions were added to neutralize the charge and bring the overall ionic strength to that of physiological saline (150 mM). Each system was then minimized by steepest descents until the maximum force was below 1000 kJ mol^−1^ nm^−1^. The systems were equilibrated at 310 K (37 °C) in the NVT ensemble for 100 ps followed by equilibration in the NPT ensemble at the same temperature at 1.0 bar pressure for another 100 ps. MD production runs for each system were then carried out in the NPT ensemble for an initial 150 ns. Inspection of the Cα RMSD matrix of each simulation suggested that both forms had equilibrated by 30 ns. Consequently, four additional 70 ns simulations with different initial seeds were run starting from the 30 ns frame of each simulation to give six independent simulations for each form. The final 120 ns of each initial simulation was merged with the final 68 ns of the four subsequent simulations to create one merged 460 ns trajectory for each fragment, and these merged simulations were subsequently analyzed and compared.

During minimization, equilibration, and simulation, all bonds involving hydrogen atoms were constrained using the LINCS algorithm [71]. A 2 fs time step was used in all simulations; the temperature was held constant using a velocity-rescaling algorithm [72], while the pressure was maintained using the C-rescale algorithm [73] with a 2 ps time constant. Long-range electrostatic interactions were calculated using the Particle-mesh Ewald scheme [74,75], and the Van der Waals and Coulomb interactions were truncated at 1.2 nm.

Clustering analysis was carried out using the GROMOS algorithm [76] with RMSD cutoffs of 0.05, 0.07, 0.10, 0.13, and 0.15 nm. The results of the 0.10 nm cutoff were judged to be the most informative. PCA was carried out on the Cα atoms of residues 581–591, 613–627, 1661–1670, and 2128–2138, which take part in the interface between all three domains. All additional analyses described in the Results, Section 2 were carried out using programs implemented in the GROMACS suite. All plots were created using R [77], and all trajectory files were converted to the PDB format and visualized using PyMOL [67].

### 4.2. Cloning of the hRyR2^1–655^ Fragment and Site-Directed Mutagenesis

A c-DNA of the *h*RyR2 N-terminal fragment containing residues 1–655 (*h*RyR2^1–655^) was amplified using a standard PCR protocol with Phusion^®^ DNA polymerase (Finnzymes, Espoo, Finland): initial denaturation at 95 °C for 2 min followed 30 cycles of denaturation at 95 °C for 30 sec, annealing at 58 °C for 60 sec, polymerization at 72 °C for 2 min, and final polymerization at 72 °C for 5 min (after Borko et al. [49]). An uncleavable 6× His-Tag was fused to the N-terminus using a forward primer (Table A5). The amplified product was inserted into a pET28a vector (Novagen, Madison, WI, USA) using the NcoI and BamHI restriction sites upstream and downstream of the coding sequence according to the protocol described in Bauerová-Hlinková et al. [78]. The resulting plasmid construct, *h*RyR2^1–655·His6^·pET28a, was introduced into *Escherichia coli* TG1 cells for amplification. The integrity of the *h*RyR2^1–655^ insert was verified by DNA sequencing.

The *h*RyR2^1–655·His6^·pET28a plasmid was used as a template for the creation of the S616L point mutant. Mutant primers are listed in Table A5. Site-directed mutagenesis was performed using the Q5^®^ Site-Directed Mutagenesis Kit (New England Biolabs, Ipswich, MA, USA) according to the manufacturer’s instructions. For PCR, we used the following optimized program: initial denaturation at 98 °C for 90 s followed by 25 cycles of denaturation at 98 °C for 80 s, annealing for 20–25 s, polymerization at 72 °C for 30 s/kbp, and final polymerization at 72 °C for 4 min. The annealing temperature was 59° according to the manufacturer’s recommendation. The S616L mutant construct was verified by DNA sequencing.

### 4.3. Heterologous Expression and Purification of Wild-Type and S616L hRyR2 NTD^1–655·His6^

For heterologous expression, *h*RyR2^1–655·His6^·pET28a and its S616L mutant construct were transformed into *E. coli* BL21 (DE3) cells. Expression of recombinant *h*RyR2 fragments was induced by 10–20 μM IPTG overnight at 18 °C as described in Borko et al. [79]. After expression, bacterial cells were harvested by centrifugation (3000× *g* for 20 min at 4 °C). Pellets were washed in 1× PBS buffer, weighed, and stored at −80 °C. For fragment isolation, cells were resuspended in lysis buffer (20 mM Tris-HCl, pH 8.3, 180 mM NaCl, 0.1% Tween-20), a protease inhibitor cocktail (Sigma-Aldrich, St. Louis, MO, USA) was added, and then sonicated at 4 °C. The lysate was centrifuged (100,000× *g* for 30 min at 4 °C), and the supernatant was applied to a gravity-flow immobilized metal ion chromatography column filled with His-Select nickel affinity gel. The target fragments were eluted with 40 mM Tris-HCl (pH 8.2), 180 mM NaCl, 300 mM imidazole, 2% glycerol, 4 mM CHAPS, and 0.2% betaine. The eluted protein was further purified using size-exclusion chromatography (SEC) on a Superdex 200 10/300 GL column (GE Healthcare, Chicago, IL, USA) pre-equilibrated with running buffer (40 mM Tris-HCl, pH 8.2, 150 mM NaCl, 2% glycerol, 4 mM CHAPS, and 0.2% betaine). Purification was conducted at 8 °C with a flowthrough of 0.4 mL/min. SEC molecular weights were calibrated using BSA (66.5 kDa), Conalbumin (75.0 kDa), and aldolase (158 kDa; Cytiva, Marlborough, MA, USA); BSA eluted at 14.0 mL, Conalbumin at 14.4 mL, and aldolase at 12.8 mL. Fractions with the target protein were pooled and concentrated to ≈3 mg/mL using an Amicon^®^ Ultra-10K (Millipore, Burlington, MA, USA). The expression, purification, and final purity of all fragments were verified using 12.5–15% SDS-PAGE. Protein concentration was measured using a NanoDrop 2000c (Thermo Fisher Scientific, Waltham, MA, USA). All chemicals were from Sigma-Aldrich unless noted otherwise.

### 4.4. LC–MS/MS Analysis

Samples (20 μL; 12 μg of protein per sample) were filled with 100 mM Tris-HCl pH 7.8 to a final volume of 100 μL. Samples were reduced by adding 5 mM dithiothreitol (30 min, 60 °C). They were subsequently alkylated in the presence of 15 mM iodoacetamide (20 min, room temperature in the dark). The modification reaction was quenched by the addition of 10 mM dithiothreitol. Next, the SP3 protein cleanup protocol with Sera-Mag Carboxylate SpeedBeads (Cytiva) was used as described in Huges et al. [80]. Washed magnetic beads were added to samples in the ratio of 10 μg beads to 1 μg protein. For protein digestion, 0.5 μg of modified sequencing grade trypsin (Promega, Madison, WI, USA) and 0.5 μg of sequencing grade chymotrypsin (Promega) were used, and the samples were incubated overnight at 37 °C and 30 °C, respectively. The reaction was stopped by 0.5% trifluoroacetic acid.

For LC–MS analysis, 0.5 μg of peptides per sample was loaded onto a nanotrap column (PepMap100 C18, 300 μm × 5 mm, 5 μm particle size, Dionex, Sunnyvale, CA, USA) and separated with an EASY-Spray C18 analytical column having an integrated nanospray emitter (75 μm × 500 mm, 2 μm particle size, Thermo Fisher Scientific, Waltham, MA, USA) mounted into an EASY-Spray ion source. Peptides were separated using an UltiMate 3000 RSLCnano system (Dionex, Sunnyvale, CA, USA) in 60 min gradient 3–43% acetonitrile with 0.1% formic acid. MS/MS data were acquired on an Orbitrap Elite mass spectrometer (Thermo Fisher Scientific) operating in data-dependent acquisition (DDA) mode using a Top15 mode for precursor ion selection. Fragments were obtained by HCD fragmentation [81]. Datasets were processed by MaxQuant (version 1.6.17) [82] with the built-in Andromeda search engine. The specific parameters for searching were carbamidomethylation (C) as a permanent modification, oxidation (M) as variable modifications, and up to 2 tryptic miscleavages. The search was performed against the protein database *Escherichia coli* (strain K12) (K12/MG1655/ATCC 47076; Uniprot UP000000625), downloaded on 26.4.2024, and against a recombinant database containing RyR_WT and mutant sequences.

### 4.5. CD Spectroscopy

The CD spectra of wild-type and S616L mutant *h*RyR2^1–655^ fragments were recorded at increasing temperature between 4 and 50 °C on an Aviv 215 spectropolarimeter in 0.01 and 0.02 cm quartz cuvettes from 260 to ≤190 nm (0.2 nm intervals, 3–5 s per point, 1 nm bandwidth, dynode voltage < 500 V). Samples were dialyzed to 150 mM NaF, 40 mM Tris-SO_4_, pH 8.3, containing 0.3% betaine, 4 mM CHAPS, and 2% glycerol and spun at 14,000× *g* for 30 min prior to measurement. Protein concentrations (1–2.5 mg/mL) were determined from absorption spectra recorded on a Beckman DU800 photometer with an $\epsilon_{280}$ extinction coefficient calculated from the amino-acid composition [83]. Secondary structure estimates were derived using BeStSel [84] (https://bestsel.elte.hu/index.php, accessed on 2 October 2026) with normalized RMS deviations between 0.006 and 0.025.

### 4.6. Nano-Differential Scanning Fluorimetry (nanoDSF) Measurements

The thermal denaturation of wild-type and S616L mutant *h*RyR2 NTD^1–655·His6^ fragments was monitored using nanoscale differential scanning fluorimetry (nanoDSF) [50]. NanoDSF measurements were performed using a Prometheus NT.48 (NanoTemper Technologies, Munich, Germany) in a buffer containing 40 mM Tris 8.3–8.5, 150 mM NaCl, 2% glycerol, 0.2% betaine, 4 mM CHAPS at protein concentrations of 0.8–2 mg/mL range as determined by NanoDrop 2000c using an $\epsilon_{280}$ extinction coefficient of 75,860 M^−1^ cm^−1^. To measure the denaturation, capillaries were filled with ≈12 μL of sample, and temperature profiles were recorded from 20 to 85 °C with a ramping speed of 1 °C per minute. T_m_ was determined from the inflection point of a plot of temperature versus the 350:330 nm fluorescence ratio. All measurements were repeated at least three times.

## 5. Conclusions

In conclusion, we employed molecular dynamics simulation to analyze the effect of the S616L mutation on a nearby three-way interdomain junction in *h*RyR2. The results suggested that the effect of this mutation is to slightly stabilize the area immediately around the mutation site, thereby causing position of the NT-helix to shift, weakening the NSol–BSol interface, which promotes the formation of a different interface conformation while increasing the conformational flexibility of the interface area and hindering correct formation of the three-way junction, potentially resulting in partial domain unzipping. We also expressed, purified, and biochemically characterized two fragments of the *h*RyR2 comprising the NTD, one wild type and the other containing the S616L mutation. CD-spectroscopy and nanoDSF both showed that this mutation slightly increases the thermal stability of the fragment, which is consistent with the molecular dynamics results.

## Figures and Tables

**Table 1:** *h*RyR2 domains used in fragment construction and interface loops used in analysis.

Domain or Sub-Domain	Residues
NSol	409–647
JSol	1641–1763, 1777–1854, 1889–2010
BSol	2053–2364, 2377–2444, 2455–2476
**Interface Loop**	**Residues**
NT-helix	581–591
Mut-helix	613–627
JSol-helix	1661–1670
BSol-loop	2128–2138

## Data Availability

The raw data supporting the conclusions of this article will be made available by the authors on request.

## Supplementary Materials

The following supporting information can be downloaded at https://www.mdpi.com/article/10.3390/ijms272110052/s1.

## Abbreviations

The following abbreviations are used in this manuscript:

*h*RyR2	Human ryanodine receptor 2
CPVT1	Catecholaminergic polymorphic ventricular tachycardia type 1
*h*RyR2 NTD^1–655·His6^	Wild-type full-length N-terminal domain of human ryanodine receptor 2
NTD	N-terminal domain
NanoDSF	Nano differential scanning fluorimetry
CD	Circular dichroism

## Appendix A

**Table A1:** H-bonds between BSol-loop and Mut-helix. Difference is S616L Presence − WT Presence.

H-Bond	WT Presence	S616L Presence	Difference
val625n—cys618o	0.9035	0.8928	−0.0108
cys620n—val623o	0.9219	0.9082	−0.0138
arg626n—arg2133o	0.2213	0.6863	0.4651
ala624n—ser2131o	0.6153	0.4547	−0.1606
arg2133n—ala624o	0.9466	0.8679	−0.0787

**Table A2:** H-bond differences in interface loops. Those also appearing in Table A1 are in italics; those forming part of the α-helix surrounding the S616L mutation site are in bold. Difference is S616L Presence − WT Presence.

H-Bond	WT Presence	S616L Presence	Difference
*arg626n—arg2133o*	*0.2213*	*0.6863*	*0.4651*
arg2133ne—asn587od1	0.3310	0.2046	−0.3106
arg626ne—glu2138oe1	0.0047	0.3146	0.3099
ile589n—ala585o	0.3750	0.0809	−0.2941
arg626ne—glu2138oe2	0.0052	0.2678	0.2626
ala1666n—ala1663o	0.4349	0.6878	0.2529
ser582og—ala585n	0.5605	0.7895	0.2290
ser2131n—ser2128o	0.4887	0.2727	−0.2160
val2132n—leu2129o	0.3047	0.4845	0.1798
his621ne2—glu581o	0.1778	0.0005	−0.1773
**cys618n—leu614o**	**0.7070**	**0.8798**	**0.1728**
arg626nh2—glu2138oe1	0.0012	0.1699	0.1688
arg626nh2—glu2138oe2	0.0028	0.1642	0.1614
*ala624n—ser2131o*	*0.6153*	*0.4547*	*−0.1606*
asn587n—pro583o	0.1171	0.2766	0.1594
**val619n—cys615o**	**0.8236**	**0.9767**	**0.1531**
glu2138n—glu2138oe1	0.3499	0.2219	−0.1281
arg626nh1—ala1666o	0.5958	0.7184	0.1226
ala1666n—ser1662o	0.1658	0.0520	−0.1138
asn587nd2—pro583o	0.0170	0.1250	0.1080
ala585n—ser582o	0.1597	0.0563	−0.1035
cys618n—cys615o	0.1119	0.0165	−0.0955
arg2133nh2—asn587od1	0.1109	0.0176	−0.0933
asn1669n—cys1665o	0.8603	0.7678	−0.0925
asn1669nd2—val1664o	0.5396	0.4473	−0.0923
**leu617n—val613o**	**0.8733**	**0.9619**	**0.0886**
glu584n—glu584oe1	0.0076	0.0908	0.0832
ser582og—glu584n	0.1247	0.0418	−0.0829
*arg2133n—ala624o*	*0.9466*	*0.8679*	*−0.0787*
his1670n—gly1668o	0.1471	0.0687	−0.0785
ile588n—glu584o	0.2812	0.2055	−0.0758
leu586n—ser582o	0.0580	0.1329	0.0750
leu586n—pro583o	0.2349	0.1603	−0.0746
lys590nz—ile588o	0.2192	0.1568	−0.0624
ile588n—ala585o	0.3914	0.3312	−0.0602
val623n—cys620o	0.8422	0.8969	0.0547
asn587n—glu584o	0.4757	0.4241	−0.0516
gly2135n—glu2138oe2	0.2761	0.2297	−0.0464
glu584n—glu584oe2	0.0105	0.0553	0.0448
ser582og—glu584oe1	0.0036	0.0482	0.0446
gly2135n—glu2138oe1	0.3106	0.2718	−0.0387
glu2138n—glu2138oe2	0.2515	0.2149	−0.0367
arg2133nh2—glu584oe2	0.0358	0.00002	−0.0358
his621ne2—ser582o	0.0274	0.0600	0.0326
cys1665n—tyr1661o	0.5135	0.5431	0.0296
val2132n—ser2128o	0.0295	0.0590	0.0295
leu617n—leu614o	0.0310	0.0026	−0.0284
lys590nz—asn587o	0.0308	0.0029	−0.0280
his1670ne2—glu2137oe2	0.0013	0.0285	0.0272
ser2131og—ser2128o	0.0439	0.0229	−0.0210
arg2133nh1—leu2130o	0.0003	0.0180	0.0177
arg2133nh1—val2132o	0.0032	0.0201	0.0168
met2134n—val2132o	0.0071	0.0237	0.0166
arg626nh1—leu1667o	0.0900	0.0737	−0.0164
arg626nh2—leu1667o	0.9818	0.9657	−0.0161
his621ne2—glu581oe1	0.0143	0.0004	−0.0139
*cys620n—val623o*	*0.9219*	*0.9082*	*−0.0138*
ser582og—glu584oe2	0.0030	0.0158	0.0129
glu591n—glu591oe2	0.0335	0.0222	−0.0113
*val625n—cys618o*	*0.9035*	*0.8928*	*−0.0108*

**Table A3:** Top 40 isolated H-bonds.

H-Bond	Frames	Presence	Type
asn587nd2—arg2133o	1467	0.03189	wt only
arg626nh2—ser2128og	1289	0.02802	wt only
arg2133nh1—glu584oe1	1275	0.02772	wt only
his1670ne2—ser2128og	1243	0.02702	wt only
arg2133nh2—glu584oe1	1204	0.02617	wt only
arg2133nh1—glu584oe2	796	0.01730	wt only
val619n—ser616o	705	0.01533	wt only
ser616og—val613o	593	0.01289	wt only
arg2133nh2—cys620o	521	0.01133	wt only
arg2133nh2—his621nd1	279	0.00607	wt only
ser616n—val613o	211	0.00459	wt only
leu616n—val613o	207	0.00450	mut only
val619n—leu616o	140	0.00304	mut only
ser616og—ser616o	130	0.00283	wt only
arg2133ne—val2132o	122	0.00265	mut only
arg2133nh2—leu2130o	86	0.00187	mut only
arg626ne—arg2133o	78	0.00170	mut only
arg2133nh2—val2132o	68	0.00148	mut only
arg626nh2—ser2128o	67	0.00146	wt only
his1670n—glu2138oe2	64	0.00139	mut only
arg2133ne—pro583o	63	0.00137	wt only
arg2133n—arg2133ne	43	0.00093	mut only
arg2133nh2—ser2131o	43	0.00093	mut only
his1670n—glu2138oe1	32	0.00070	mut only
asn587nd2—ser627og	30	0.00065	wt only
cys620n—ser616o	30	0.00065	wt only
arg626nh1—ala624o	22	0.00048	mut only
glu2138n—glu2137oe2	22	0.00048	mut only
lys2136nz—glu591oe2	22	0.00048	wt only
lys2136nz—glu591oe1	18	0.00039	wt only
cys620n—leu616o	17	0.00037	mut only
glu2138n—glu2137oe1	17	0.00037	mut only
arg2133nh2—ala624o	14	0.00030	mut only
arg626nh2—arg2133o	12	0.00026	mut only
ser616og—leu617n	12	0.00026	wt only
ser627n—glu2138oe1	12	0.00026	wt only
ser2128og—gly1668o	12	0.00026	wt only
arg2133nh2—leu586o	11	0.00024	mut only
asn587nd2—gly2135n	11	0.00024	wt only
ser627og—glu591oe2	8	0.00017	mut only

**Table A4:** Possibly pathogenic mutations in and around the region 545–640.

Mutation	ClinVar Classification	Variation ID	Reference
R545C	Uncertain	3071014	
R545G	Uncertain	1358981	
N547I	Uncertain	3380782	[55]
N547K	Conflicting	201219	unpublished
A549V	Pathogenic	1330215	[22,56,57]
L555V	Uncertain	3069621	[63]
S560G	Uncertain	956086	
L565Q	Uncertain	2774249	
E566D	Uncertain	1171374	
A567P	Uncertain	691812	[58,59]
G570A	Uncertain	922097	
I571T	Uncertain	3572681	
I571N	Uncertain	296712	
H576R	Uncertain	201376	
E591Q	Uncertain	2037057	
E591G	Uncertain	2962545	
E591V	Uncertain	2454011	
I594T	Uncertain	3690566	
S596F	Uncertain	3385676	
I597F	Uncertain	2704538	
L600V	Uncertain	2763239	
L600H	Uncertain	2141934	
H604R	Uncertain	450361	
G605V	Pathogenic	2737123	unpublished
R606S	Uncertain	1709321	
N607Y	Uncertain	422186	
L611P	Uncertain	201224	
C615F	Uncertain	945913	
S616L	Pathogenic	180486	[33]
G622R	Uncertain	923549	[60,61]
A624T	Uncertain	947851	
A624V	Uncertain	238226	[62]
R626C	Uncertain	3623525	
S627C	Uncertain	2111129	
N628H	Uncertain	3073541	
I632V	Uncertain	2804904	
D634G	Uncertain	3073457	
L636I	Uncertain	1498489	
R640G	Uncertain	279589	[62]
Mutations in surrounding domains			
V1664F	Uncertain	201253	[65,66]
V1664D	Uncertain	238236	
L1667P	Uncertain	924339	
N1669S	Uncertain	165099	[64]
L2129P	Uncertain	1753196	
V2132M	Uncertain	3385762	
E2137K	Uncertain	3075091	
E2138K	Likely pathogenic	532366	unpublished

**Table A5:** Primers for the cloning and site-directed mutagenesis of the *h*RyR2^1–655^ fragment. All primers are given in the 5′ to 3′ orientation.

Cloning Primers	Primer Sequence ^1^
*h*RyR2^1–655·His6^·pET28a	F: GGCCATGGGCCATCATCATCATCATCATGCCGATGGGGGCGAGGGCGAAGAC
	R: CCGGATCCTTACATGCTGCTGACATGGTTC
**Mutation primers**	
S616 (TCA) ⟶ L (TTA)	F: TGTCTTGTGCTTACTCTGTGTTTG
	R: TCCAGAACCTTGTGATTTC

^1^ F—forward, R—reverse.
