# Supplementary material for: The Effect of S616L, a CPVT1-Associated Mutation, on the Stability and Structure of Human Cardiac RyR2
Source: Int J Mol Sci. 2026 Nov 9;27(21):10052. doi: 10.3390/ijms272110052 (PMC13763708; doi:10.3390/ijms272110052)
Supplement: Supplementary file 1 [file ijms-27-10052-s001.zip › ijms-4533538-supplementary.pdf]

Supplementary Material

To accompany

The effect of S616L, a CPVT1 associated mutation, on the stability and structure of human cardiac RyR2

Vladena Bauerová-Hlinková, Mário Benko, Ľubica Šutoová, Konrad Beck, Jakub Novak, Jana Bellová, Peter Baráth and Jacob A. Bauer

Submitted to the *International Journal of Molecular Science*

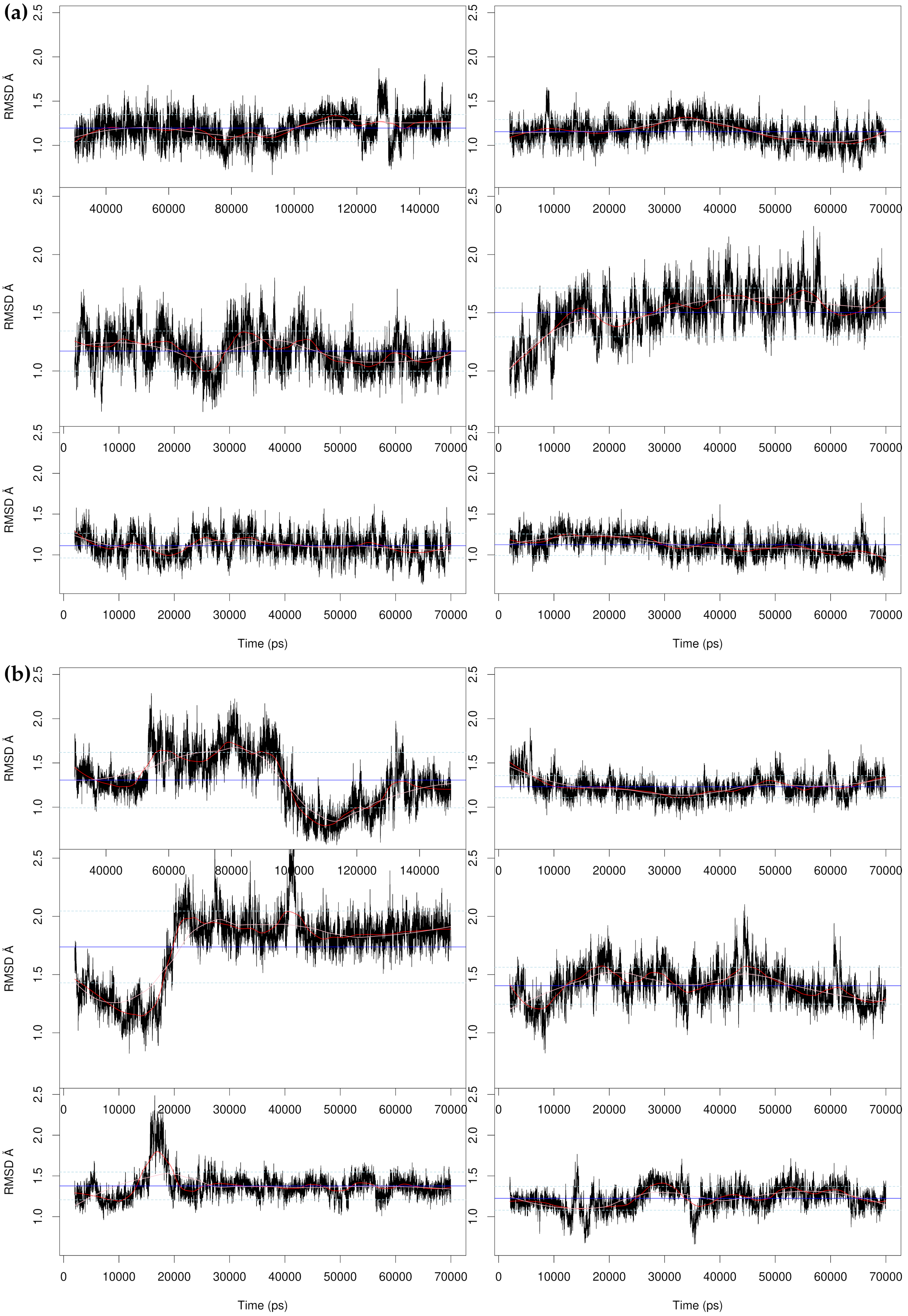

Figure S1: Plots of RMSD of the starting structure versus each of the individual trajectories that were combined to create the merged trajectories that were analyzed in this study. Shown are (**a**) the wild-type RMSD plots and (**b**) the S616L mutant RMSD plots.

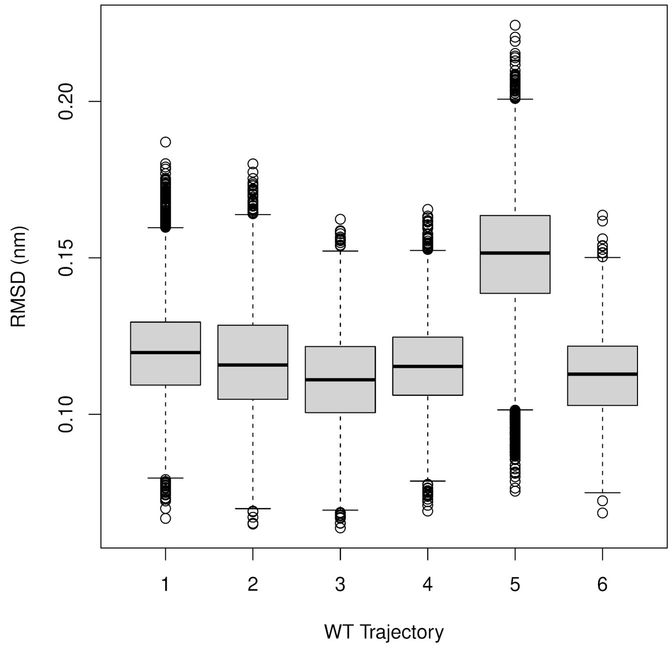

Figure S2: Boxplot showing the root-mean-squared deviation (RMSD) of six wild-type trajectories with respect to the initial structure.

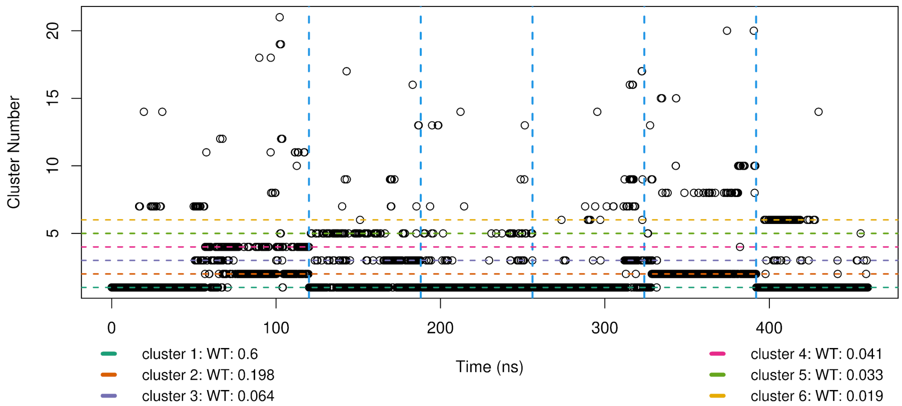

Figure S3: A plot of trajectory time-stamp versus cluster number for a merged trajectory consisting of all six wild-type trajectories. The blue dashed lines mark the boundaries of the individual trajectories. The six largest clusters are marked out using differently colored horizontal dashed lines. The figure legend shows the fraction of the merged trajectory occupied by each cluster.

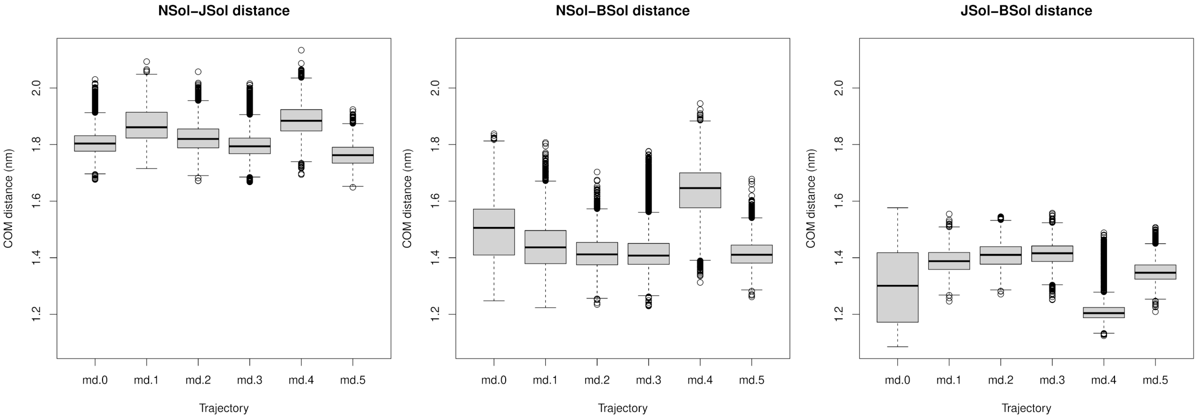

Figure S4: Three boxplots showing the distances between the centers-of-mass (COM) of the Cα atoms of the individual interfae loops in the six wild-type trajectories.
